# Quality of care index for acute lymphoblastic leukaemia at global, regional, and national levels: a systematic analysis of the global burden of disease from 1990 to 2021

**DOI:** 10.7189/jogh.16.04044

**Published:** 2026-04-03

**Authors:** Fei Wei, Lu Dai, Qiuxiang Tan, Xuan Zhou, Qingxiang Huang

**Affiliations:** Department of Hematology, The Affiliated Jiangning Hospital of Nanjing Medical University, Nanjing, Jiangsu, China

## Abstract

**Background:**

Acute lymphoblastic leukaemia (ALL) is a highly heterogeneous hematologic malignancy. Despite continuous optimisation of treatment strategies, significant disparities in care quality persist globally. This study systematically analysed trends in global ALL care quality from 1990 to 2021, focusing on variations in distinct countries, regions, age groups, and genders.

**Methods:**

Acute lymphoblastic leukaemia burden data (1990–2021) were from the Global Burden of Diseases Study (GBD) 2021. Care quality was measured utilising the quality-of-care index (QCI), which integrated incidence rate, prevalence rate, mortality, and disease burden ratio through principal component analysis. Gender disparities in QCI were assessed via the gender disparity ratio (GDR). Estimated annual percentage change (EAPC) was applied to analyse temporal trends in QCI and GDR.

**Results:**

The QCI of global ALL increased from 31.35 (95% uncertainty intervals (UI) = 31.21, 31.45) in 1990 to 58.46 (95% UI = 58.36, 58.58) in 2021, with an EAPC of 2.27 (95% confidence intervals (CI) = 2.16, 2.38). GDR increased from 0.96 in 1990 to 1.00 in 2021 (EAPC = 0.18; 95% CI = 0.15, 0.22). In 2021, the high sociodemographic index (SDI) region achieved a QCI of 90.75 (95% UI = 90.72, 90.85), whereas the low-SDI region lagged at 14.10 (95% UI = 13.97, 14.16). Gender disparities were minimal in regions with high SDI but persisted in those with low SDI, where females received inferior care quality and disparities widened. Among children aged 0–5 years, QCI was the highest, while among elderly populations, it scored lower.

**Conclusions:**

Global ALL care quality improved from 1990 to 2021, with reduced gender disparities. However, inequities across regions, ages, and genders remain. Future interventions should target low-SDI regions and elderly patients through optimised resource allocation to promote equitable global ALL care development.

Acute lymphoblastic leukaemia (ALL) is a highly heterogeneous hematologic malignancy marked by the abnormal development and infiltration of immature lymphocytes in the bone marrow, blood, and other extramedullary sites [1]. According to global statistics, there were 103 727 new ALL cases in 2021, with 71 221 deaths, and the incidence was generally higher in males than in females [2]. Although ALL is more common in children, it can also affect adults [3]. The treatment efficacy and prognosis of ALL largely depend on the quality of nursing care. The 5-year survival rate of ALL children receiving treatment in high-income countries has exceeded 90%, while in low- and middle-income countries, it is only 52.9% [4,5].

The WHO describes quality of care as the extent to which health services provided to populations increase the probability of achieving optimal health outcomes [6]. Therefore, objective measurements of health outcomes can serve as critical indicators for evaluating the quality of care across health care systems [7]. However, in many low- and middle-income countries, tumour registration systems are not perfect, and clinical outcome data are scarce [8]. Traditional single indicators, such as incidence and mortality, cannot fully reflect the true state of medical quality. The Healthcare Access and Quality (HAQ) index is constructed based on age-standardised mortality rates of 32 preventable or treatable diseases to reflect the comprehensive ability of a country’s health care system to reduce avoidable deaths [9]. However, the HAQ index mainly focuses on the overall health care system level and is difficult to reflect the care quality and management effectiveness of specific diseases.

As an innovative composite evaluation tool, the Quality of Care Index (QCI) integrates multiple indicators of specific diseases through principal component analysis, including the ratio of mortality to incidence rate, the ratio of disability adjusted life years (DALYs) to morbidity, the ratio of morbidity to incidence rate, and the ratio of years of life lost (YLL) to years of disability (YLD). Compared to a single indicator, QCI can more comprehensively reflect the overall effectiveness of disease management and adopt a standardised scoring system of 0–100, which facilitates horizontal comparison between different countries and regions [10]. Although previous studies have explored QCI for different types of leukaemia and found significant regional differences, there is still a lack of in-depth analysis on the gender differences in the quality of ALL care in different regions [11].

Against this background, this study utilised the Global Burden of Diseases, Injuries, and Risk Factors Study (GBD) database from 2021 to systematically analyse the trends in care quality for ALL worldwide. The gender disparity ratio (GDR) was used to evaluate the gender differences in care quality in different regions [12]. This study focused on the changing characteristics of QCI and GDR in different countries, regions, and age groups. The findings revealed the current state of global ALL care quality and gender disparities, providing evidence-based insights to support efforts toward health equity for ALL patients worldwide.

## METHODS

This study strictly followed the GRADROP guidelines to ensure research rigor [13] (Table S1 in the **Online Supplementary Document****)**.

### Data sources

Acute lymphoblastic leukaemia burden data were from GBD 2021 [14]. Reported estimates were calculated as the mean of 1000 draws from the distribution, with 95% uncertainty intervals (UIs) derived as the 2.5th and 97.5th percentiles across all draws [15,16].

We extracted data on ALL incidence, prevalence, YLLs, deaths, DALYs, YLDs, and corresponding crude rates and age-standardised rates stratified by gender and age in the world, five regions with different socio-demographic index (SDI), 21 areas, and 204 countries/territories from 1990 to 2021. The latest SDI estimates from GBD, scaled from 0 to 1, were used as a composite indicator of national development status [17]. 204 countries/territories were categorised into low-SDI, low-middle-SDI, middle-SDI, high-middle-SDI, and high-SDI regions according to quintiles of SDI [18].

### Disease definition

In the GBD 2021 cause list, ALL corresponds to codes in the ICD-10 revision, including C91.0–C91.02, C91.2–C91.32, and C91.6–C91.62.

### Quality of Care Index

In this study, QCI was used to evaluate the quality of care for global ALL, which indirectly measured the quality of care and overall disease management effectiveness at the macro level through disease outcomes. A previous study has confirmed QCI applicability in the field of leukaemia [11]. QCI is a comprehensive index, which integrates the following four ratios through principal component analysis: the ratio of mortality to incidence rate (reflecting the survival after diagnosis and the effect of nursing intervention), the ratio of DALYs rate to prevalence rate (measuring the degree of disease burden relative to prevalence rate and reflecting the efficiency and effectiveness of medical interventions), the ratio of YLL rate to YLD rate (representing the balance between disease mortality and disability and reflecting health care’s focus on extending lifespan); and the ratio of morbidity to incidence rate (indicating the management and control degree of new cases in the population. The first principal component extracted from these four ratios was QCI (Figure S1–4 in the **Online Supplementary Document**). QCI was subsequently rescaled to the range of 0 to 100 [19]. The higher QCI value indicated the better overall disease outcome and burden management performance of the ALL nursing system. This study used bootstrap to conduct 1000 repeated samplings, with the 2.5th and 97.5th percentiles taken as the 95% uncertainty intervals for the observation.

To assess gender disparities at a macro level, the GDR was the result of female QCI divided by male QCI, with GDR>1 indicating higher female care quality than male, GDR<1 indicating higher male care quality than female, and GDR ≈ 1 indicating minimal disparity [12].

### Statistical analysis

We used descriptive analysis to analyse the QCI and GDR of ALL in the global and five SDI regions from 1990 to 2021, and estimated annual percentage change (EAPC) to display time trends. The natural logarithms of QCI or GDR were fitted to the year through a regression model, where ε indicated error item. The calculation formulas of the regression model and EAPC were as follows:

ln(QCI) or ln(GDR) = α + β × year + ε

EAPC = 100 × (exp(β) − 1)

Trends were determined as increasing trend (EAPC and 95% confidence interval (CI) lower bound >0), decreasing trend (EAPC and 95% CI upper bound <0), and stable trend (95% CI includes 0) [20]. We graphed maps to display 1990 and 2021 QCI/GDR for 204 countries/territories. In addition, to evaluate the trend of care quality in different age groups, we divided the data into 20 age groups with 5-year intervals (<5 years, 5–9 years, ..., 95+ years), and conducted trend analysis on QCI and GDR of various age groups worldwide in 1990 and 2021. Finally, using equal weight calculation, removing ratios one by one, and min-max normalisation of PCA, QCI was calculated for sensitivity analysis to verify the robustness and reliability of the results. All analyses were processed using *R,* version 4.3.0. (R Foundation for Statistical Computing, Vienna, Austria).

## RESULTS

### Temporal trends in global ALL care quality, 1990–2021

From 1990 to 2021, global ALL care quality demonstrated significant improvement, with the QCI increasing from 31.35 in 1990 (95% UI = 31.21, 31.45) to 58.46 (95% UI = 58.36,58.58), with EAPC as 2.27 (95% CI = 2.16, 2.38). The increase was mainly due to the overall improvement of the ratio of mortality to incidence rate, the ratio of DALY to morbidity, and the ratio of YLL to YLD (Table S2 in the **Online Supplementary Document**), reflecting improvements in reducing ALL mortality and disease burden. Concurrently, the GDR rose from 0.96 in 1990 to 1.00 in 2021 (EAPC = 0.18; 95% CI = 0.15, 0.22), indicating a gradual reduction in gender-based disparities in care quality (**Table 1**).

**Table 1:** QCI and GDR for acute lymphoid leukaemia in 1990 and 2021 across global and 5 SDI regions, and their EAPC

Location	QCI			GDR		
	**QCI (95% CI), 1990**	**QCI (95% CI), 2021**	**EAPC (95% CI)**	**1990**	**2021**	**EAPC (95% CI)**
Global	31.35 (31.21, 31.45)	58.46 (58.36, 58.58)	2.27 (2.16, 2.38)	0.96	1.00	0.18 (0.15, 0.22)
Low SDI	8.01 (7.88, 8.05)	14.10 (13.97, 14.16)	1.75 (1.64, 1.87)	1.02	0.93	−0.22 (−0.29, −0.16)
Low-middle SDI	10.35 (10.23, 10.40)	24.34 (24.22, 24.44)	2.88 (2.80, 2.97)	0.76	0.97	0.97 (0.91, 1.03)
Middle SDI	18.28 (18.15, 18.35)	57.47 (57.35, 57.59)	4.10 (3.93, 4.28)	0.99	1.01	0.05 (0, 0.09)
High-middle SDI	38.11 (37.99, 38.21)	82.68 (82.62, 82.77)	2.75 (2.57, 2.93)	0.99	1.01	0.07 (0.04, 0.11)
High SDI	75.82 (75.75, 75.93)	90.75 (90.72, 90.85)	0.59 (0.51, 0.66)	1.02	1.00	−0.04 (-0.06, −0.02)

CI – confidence interval, EAPC – estimated annual percentage change, GDR – gender disparity ratio, QCI – quality of care index, SDI – sociodemographic index

**Figure 1:**
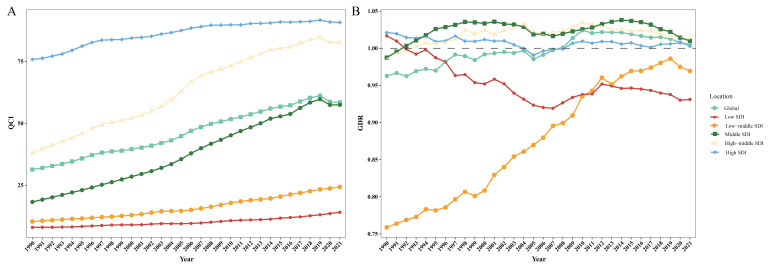
Trends in QCI (**Panel A**) and GDR (**Panel B**) for global and 5 SDI regions in ALL from 1990 to 2021. GDR – gender disparity ratio, QCI – quality of care index, SDI – sociodemographic index.

Regional analysis revealed a favourable linkage between SDI levels and QCI. In 2021, QCI was the highest in high-SDI regions (QCI = 90.75; 95% UI = 90.72, 90.85), while the lowest was in low-SDI regions (QCI = 14.10; 95% UI = 13.97, 14.16). All five SDI regions showed upward QCI trends during 1990–2021. The middle-SDI region demonstrated the most pronounced improvement (EAPC = 4.10; 95% CI = 3.93, 4.28) (**Figure 1**, Panel A, **Table 1**; Table S3 in the **Online Supplementary Document**).

Except for high-SDI areas, all regions had gender disparities that persisted in 2021. Females experienced marginally better care quality in high-middle-SDI and middle-SDI regions (GDR = 1.01), while males in low-middle-SDI (GDR = 0.97) and low-SDI regions (GDR = 0.93) had advantages. Between 1990 and 2021, the GDR of low-SDI regions showed a downward trend, gradually decreasing from 1.02 in 1990 to 0.93 in 2021 (EAPC = −0.22; 95% CI = −0.29, −0.16), and gender differences widened. The GDR in the middle- and low-SDI regions showed an upward trend (1990 = 0.76; 2021 = 0.97), and the difference in care quality between genders gradually narrowed (**Figure 1**, Panel B, **Table 1**; Table S4 in the **Online Supplementary Document**).

### Regional disparities in Global ALL Care Quality, 1990–2021

**Figure 2:**
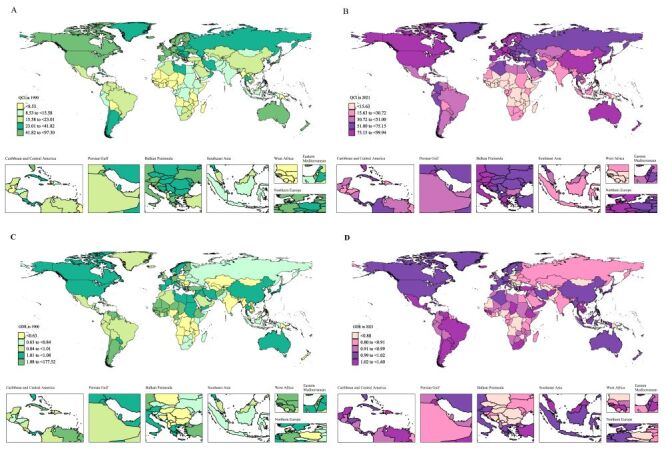
Maps of QCI and GDR for ALL in 204 countries and regions in 1990 and 2021. **Panel A**. QCI in1990. **Panel B**. QCI in 2021. **Panel C**. GDR in 1990. **Panel D**. GDR in 2021. ALL – acute lymphoblastic leukaemia, GDR – gender disparity ratio, QCI – quality of care index.

Substantial disparities in ALL care quality were observed across global regions. In 2021, Western Europe, high-income Asia Pacific, Australasia, and high-income North America ranked as the top four regions with high QCI (all >85), maintaining the same positions held in 1990. Conversely, Western sub-Saharan Africa, Oceania, and Eastern sub-Saharan Africa demonstrated the lowest QCIs (<15), replicating their 1990 rankings. At the national level, Monaco achieved the highest QCI of ALL in 2021, with Switzerland and Spain following. In stark contrast, Chad, Kiribati, and Niger recorded the lowest QCIs (**Figure 2**, Panels A–B, **Table 2**; Table S5 in the **Online Supplementary Document**).

**Table 2:** The three countries with the highest and lowest QCI and GDR for acute lymphoid leukaemia in 1990 and 2021

Measure	The top three countries	Bottom three countries
**QCI in 1990**	Monaco (97.30)	Guinea (0.55)
	San Marino (96.41)	Nigeria (0.26)
	Switzerland (94.39)	Niger (0)
**QCI in 2021**	Monaco (99.94)	Niger (6.96)
	Switzerland (99.80)	Kiribati (6.68)
	Spain (97.98)	Chad (5.23)
**GDR in 1990**	Guinea (177.52)*	Nigeria (0.20)
	Guinea-Bissau (6.52)	Mozambique (0.12)
	Mali (2.83)	Kiribati (0)
**GDR in 2021**	United States Virgin Islands (1.60)	Papua New Guinea (0.62)
	Guinea-Bissau (1.56)	Central African Republic (0.61)
	Bermuda (1.49)	North Macedonia (0.57)

GDR – gender disparity ratio, QCI – quality of care index

*The high GDR in Guinea is due to the relatively low QCI in males.

GDR of care quality was achieved as 1 in 2021 within Western Europe and high-income Asia Pacific, indicating that gender disparities in care quality were nearly eliminated in these regions. Oceania, Central Sub-Saharan Africa, and Central Asia exhibited the lowest GDRs (≤0.75), indicating that the quality of care in males was superior to that of females. Central sub-Saharan Africa and Oceania were also the two regions with the lowest GDR in 1990. At the national level, in 2021, the quality of care in countries such as Myanmar, the Netherlands, and Japan had basically reached a gender balance (GDR = 1). The most extreme female advantages in care quality emerged in the USA. Virgin Islands, Guinea, and Bermuda, while North Macedonia, the Central African Republic, and Papua New Guinea displayed the strongest male advantages in the quality of care (**Figure 2**, Panels C–D, **Table 2**; Table S6–7 in the **Online Supplementary Document**).

### Global are Quality of ALL in different age groups in 1990 and 2021

**Figure 3:**
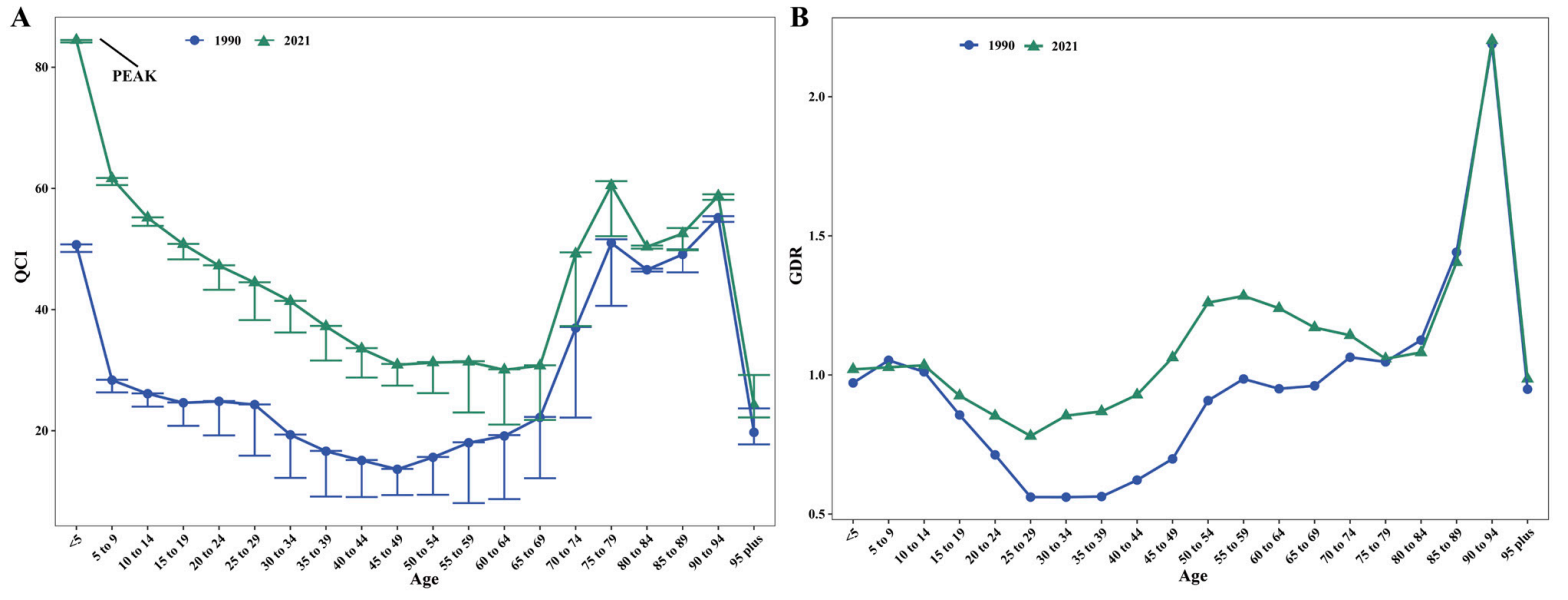
QCI (**Panel A**) and GDR (**Panel B**) for acute lymphoid leukaemia in different age groups globally in 1990 and 2021. In 2021, QCI was highest among children aged 0–5 years, GDR was lowest in the 25–29 age group, and highest in the 90–94 age group. In **Panel A**, the 95% uncertainty intervals for the 55–79 age group are relatively wide, which may be related to the small values of the DALY rate-to-prevalence ratio. GDR – gender disparity ratio, QCI – quality of care index.

**Figure 3** illustrates the QCI and GDR trends in age groups globally for 1990 and 2021. All age groups in 2021 showed higher QCIs compared to 1990, with particularly significant improvements observed in paediatric and young populations. The overall quality of nursing decreases with age, with the highest QCI (84.47, 95% UI = 84.09, 84.50) in the 0–5 age group, followed by a gradual decrease and an M-shaped fluctuation in the elderly stage (**Figure 3**, Panel A; Table S8 in the **Online Supplementary Document**).

Regarding gender disparities, while most age groups in 1990 showed better care quality for males than females, this difference improved by 2021. In 2021, the quality of nursing care for women aged 0–14 was slightly higher than that for men; The opposite was true for the population aged 15–44, with the male advantage being most pronounced between the ages of 25–29 (GDR = 0.78). The 45–94 age group showed that the quality of nursing care for women was better than that for men, reaching a peak of 2.20 in the 90–94 age group. The gender gap among people aged 95 and above became more balanced (**Figure 3**, Panel B; Table S9 in the **Online Supplementary Document**).

### Sensitivity analysis of the 2021 Global Care Quality ranking

In the case of using equal weight calculation, removing ratios one by one, and Min-Max standardisation as alternatives to PCA, the top 5 and bottom 5 countries in QCI ranking were basically consistent, indicating the robustness of the conclusions of this study (Table S10 in the **Online Supplementary Document**).

## DISCUSSION

This study systematically analysed global trends in ALL care quality from 1990 to 2021 using GBD 2021 data. Significant global improvement in the care quality of ALL was exhibited, particularly in middle-SDI regions. However, persistent disparities across regions and populations highlighted challenges in health care resource allocation and health care service accessibility.

Among different regions, the improvement of QCI was most significant in middle-SDI regions. International cooperation has played a key role in promoting the improvement of ALL care quality in these regions. For example, the collaborative project between Colombia and Dana Farber Cancer Center in the USA significantly reduced treatment-related mortality and treatment abandonment rates in children with ALL [21]. Second, the expansion of medical insurance coverage and the reform of the health care system provide institutional guarantees for improving the quality of care for ALL. The national health insurance programme Seguro Popular implemented in Mexico has effectively improved patients’ access to medical services and reduced their economic burden [22]. In addition, the ‘My Child Matters’ programme funded by Sanofi has also played an important role in improving paediatric oncology care in resource-limited countries. For example, in Paraguay, the project established satellite clinics and multidisciplinary teams, developed ‘risk of abandonment’ assessment tools, and provided early diagnosis training [23].

The global QCI for ALL is elevated, reflecting advancements in medical technology, optimised treatment strategies, and the dissemination of clinical practice guidelines [24]. The introduction of targeted therapies and immunotherapies into clinical practice in recent years has substantially improved therapeutic outcomes and care quality for ALL patients. Imatinib combined with intensive chemotherapy significantly improved 3-year event-free survival in ALL patients with Philadelphia chromosome-positive (Ph+), which is more than twice as many patients who only received intensive chemotherapy [25]. In a multicentre, phase II clinical trial, researchers verified the efficacy and safety of the therapy combining venetoclax with Azacitidine in treating relapsed or refractory T-cell ALL and lymphoblastic lymphoma, with a total effective rate of up to 88.9% [26]. In addition, immunotherapy, especially chimeric antigen receptor (CAR) T-cell therapy, holds promise in ALL treatment. Follow-up analyses from a phase I investigation showed CD19-specific CAR-T cell therapy yielded complete responses in 80% of treated adult ALL patients (n = 53), with a median overall survival of 12.9 months [27].

Although the overall quality of care for ALL was on the rise globally, there were still significant differences between different regions. The QCI of high-SDI regions reached 90.75, while that of low-SDI regions was only 14.10. Regions like Western Europe and high-income Asia Pacific regions, with high-SDI, are usually equipped with advanced medical equipment that can carry out refined diagnosis and treatment, such as molecular typing and minimal residual disease (MRD) detection, while also possessing the ability to obtain innovative treatment options (such as CAR-T and targeted drugs) [28]. In contrast, the quality of care in low-income areas such as sub-Saharan Africa is generally low, limited by a shortage of medical resources, weak infrastructure, and a severe shortage of professional nursing and clinical professionals [29]. These regions often lack advanced diagnostic tools and innovative drugs, leading to obstacles for patients in multiple aspects, such as diagnosis, treatment, and long-term management [30,31]. Furthermore, the QCI of countries such as Chad, Kiribati, and Niger was even lower than 10, reflecting their weak state in ALL care. This reality suggests that in the global health intervention strategy, priority should be given to strengthening infrastructure investment and capacity building in countries with extremely low quality of care, and promoting the accessibility of key diagnostic and treatment resources.

There were significant disparities in the quality of care for ALL among different age groups, with the 0–5 age group having the highest quality of care. The quality of care had a significant decline in the young and middle-aged population, and maintained a low level in the middle-aged and elderly population. This difference can be explained from multiple perspectives. First, the high degree of standardisation in the diagnosis and treatment of paediatric ALL, the high participation rate in clinical trials, and the well-established specialised management system [1,32] can help improve the quality of nursing and treatment outcomes. In contrast, the participation rate in clinical trials for adult and elderly ALL patients is lower, and the proportion of Ph + patients is higher, leading to poor prognosis and decreased treatment responsiveness [32,33]. For elderly patients, their clinical management is more complex. Multiple comorbidities, progressive decline in physical function, and reduced tolerance to therapeutic toxicity (such as hepatotoxicity, pancreatitis, and thrombosis) all increase the risk of treatment. Less than 50% of elderly ALL patients can truly benefit from intensified chemotherapy regimens [34]. Therefore, it is necessary to strengthen the comprehensive evaluation and continuous optimisation of clinical treatment plans for adult and elderly patients, enhance the collaboration ability of interdisciplinary teams, and thus improve the overall nursing quality and survival prognosis of this group.

This study demonstrated that the gender disparity in the quality of care for ALL was gradually narrowing globally, and by 2021, a basic balance in the quality of care between genders had been achieved. This may be related to the increasing attention to gender health equity internationally, the enhanced accessibility of women’s health services, and the promotion of gender sensitive health policies [35,36]. However, this trend exhibits significant heterogeneity in different SDI regions. The GDR in low-SDI areas decreased to 0.93 in 2021, with an EAPC of −0.22, suggesting that the quality of care for women is lower than that for men, and gender differences are continuing to widen. Due to traditional beliefs and gender bias, women in underdeveloped areas often find themselves on the margins of resource allocation and health care decision-making. For example, in southeastern Nigeria, household resources tend to prioritise ensuring that male patients have access to formal medical services [37]. Therefore, female patients are more likely to face issues such as delayed diagnosis and interrupted treatment, which exacerbates gender inequality. On the contrary, high-SDI regions, such as Western Europe, have achieved gender equality in terms of care quality due to their strong awareness of gender equality and relatively sufficient medical resources [38]. In addition, this study also found that there are complex trends in GDR changes among different age groups. In 2021, the care quality for children and middle-aged and elderly women was better than that for men, while the care quality for young and middle-aged men was generally higher. Research has shown that women of childbearing age in low-income countries face many obstacles in accessing medical services, including economic difficulties, lower education levels of themselves and their spouses, and the influence of religious factors [39]. Although the gender disparity is narrowing globally, regional and age differences remain significant, requiring continued focus on women in low-SDI regions to achieve health equity.

In response to the low-SDI areas and relatively low-QCI in elderly patients identified in this study, targeted intervention measures should be prioritised to improve the quality of ALL care. For low-SDI areas, it is necessary to strengthen the diagnosis and treatment capacity building of grassroots and regional medical institutions, enhance international cooperation, promote MRD testing to optimise treatment plans, and ensure the accessibility of key chemotherapy drugs and targeted drugs [21,40,41]. At the same time, establishing and improving a cancer registration and monitoring system can improve data integrity and accuracy of care quality assessment [42]. For elderly patients, early screening and personalised treatment strategies should be improved, and multidisciplinary joint management should be strengthened [34].

However, there are still certain limitations. First, GBD data are mainly based on model estimates rather than raw data reported by different countries, and there are disparities in the completeness and quality of data sources in different countries and regions. These factors may have a certain impact on the accuracy of the results and comparability between countries. Second, the calculation of QCI depends on data such as incidence rate, prevalence rate, mortality and DALYs, so it is inevitably affected by incomplete case registration, survival bias and differential diagnostic coverage. Third, QCI, as an indirect indicator for evaluating the quality of ALL care, cannot directly reflect differences in accessibility of health care services, completeness of disease registration, medical infrastructure, patient compliance, and social support; Meanwhile, for cancers that include both curative and palliative treatment pathways, relying solely on QCI cannot distinguish the contribution of curative treatment outcomes from palliative management. Fourth, GDR may be affected by biological factors (such as gender specific incidence rate and survival rate), so it is impossible to distinguish between nursing inequality and biological differences. Finally, due to the limitations of the GBD database, our research cannot include key dimensions such as patient satisfaction, health care staff responsiveness, and treatment reliability and effectiveness, which, to some extent, limit the multidimensional evaluation of care quality.

## CONCLUSIONS

From 1990 to 2021, the overall quality of care for ALL worldwide improved, and gender differences tended to narrow, but regional differences remained significant. Care quality in low-SDI regions was poor, and males tended to have advantages in acquiring care resources. The quality of child care is higher than that of adults, while it is relatively lower for the elderly population. In the future, targeted intervention strategies should be developed for low-SDI areas and elderly patients to promote the global balanced development of ALL nursing quality.

## Additional material


Online Supplementary Document
