# Supplementary material for: Quality of care index for acute lymphoblastic leukaemia at global, regional, and national levels: a systematic analysis of the global burden of disease from 1990 to 2021
Source: J Glob Health. 2026 Apr 3;16:04044. doi: 10.7189/jogh.16.04044 (PMC13047350; doi:10.7189/jogh.16.04044)
Supplement: Online Supplementary Document [file jogh-16-04044-s001.pdf]

Supplement to:Wei F, Dai L, Tan Q, Zhou X, Huang Q. Quality of care index for acute lymphoblastic leukaemia at global, regional, and national levels: a systematic analysis of the global burden of disease from 1990 to 2021. J Glob Health. 2026;16:04044.

```
> pca_out$var$contrib
              Dim.1    Dim.2     Dim.3     Dim.4
MIR       25.10133 23.91661 12.383209 38.598851
YLLtoYLD  25.57121 10.37786 56.647383  7.403544
DALtoPER  24.12998 42.91003 29.433535  3.526461
PERtoINC  25.19748 22.79550  1.535874 50.471144
```

Figure S1. Percent of contribution of each of four secondary variables (as entries) with different components[=dimensions] of PCA on age-standardized both-sex of acute lymphoid leukemia from 1990-2021. Abbreviations: MIR, mortality-to-incidence ratio; YLL to YLD, years of life lost rate-to-years lived with disability rate tatio; DAL to PER, disability adjusted life years rate-to-prevalence ratio; PER to INC, prevalence-to-incidence ratio.

```
> pca_out$eig
        eigenvalue percentage of variance cumulative percentage of variance
comp 1 3.799202452            94.98006131                          94.98006
comp 2 0.182450767             4.56126917                          99.54133
comp 3 0.016662056             0.41655139                          99.95788
comp 4 0.001684725             0.04211813                         100.00000
```

Figure S2. The eigenvalue of the first component was close to 4 (as the largest possibility in a 4-dimension PCA) and has grabbed 94.98% of variability and information of total data points in age-standardized both-sex population.

```
> pca_out$var$coord
              Dim.1      Dim.2       Dim.3        Dim.4
MIR       0.9765502 -0.2088924  0.04542353  0.025500676
YLLtoYLD  0.9856481  0.1376026 -0.09715255  0.011168230
DALtoPER  0.9574689  0.2798029  0.07003022 -0.007707864
PERtoINC -0.9784187  0.2039377  0.01599713  0.029159904
```

Figure S3. The slopes of each of four entry variables in each of dimensions. Abbreviations: MIR, mortality-to-incidence ratio; YLL to YLD, years of life lost rate-to-years lived with disability rate tatio; DAL to PER, disability adjusted life years rate-to-prevalence ratio; PER to INC, prevalence-to-incidence ratio.

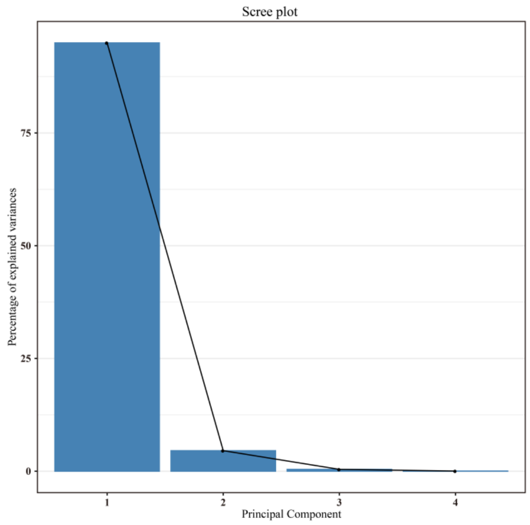

Figure S4. Scree plot from principal components analysis of acute lymphoid leukemia from 1990-2021.

Table S1. Outline of JoGH guideline items.

JoGH guideline item	Author's Response
1. Please list all papers published by each co-author in previous 3 years that were based on secondary analysis of a big data repository	None.
2. Please explain the key elements of your study design and the use of the available datasets that make your study an original scientific contribution	Based on global, regional, and national data from the GBD 1990–2021, this study developed a Quality of Care Index (QCI) for acute lymphoid leukemia (ALL) to reflect the level of disease management. A gender disparity ratio (GDR) was incorporated to quantify differences in care quality between males and females, and the care status across different age groups was also described. This study not only provides a comprehensive assessment of ALL care quality at the global, regional, and national levels but also offers scientific evidence to inform health policy and optimize resource allocation, highlighting its academic contribution and originality.
3. Please list all publications that addressed similar research questions in the same dataset and indicate where you cited them in your paper	In the last sentence of the second paragraph of the Introduction, we cited previous study that investigated the QCI for different types of leukemia and reported significant regional disparities. However, these studies did not provide an in-depth analysis of sex-specific differences in ALL care quality across regions. Our study addresses this gap by introducing the GDR to quantify differences in care quality between males and females. This allows us to systematically evaluate both regional and sex-related variations in ALL management.
4. Please explain how you addressed multiple testing through an appropriately rigorous statistical threshold and indicate this in the methods section	Since the QCI and GDR are composite descriptive indices intended to summarize care quality and sex-related differences across countries and regions, no hypothesis testing was conducted for individual countries or subgroups, and no correction for multiple testing was applied. The primary purpose of these indices is to provide a descriptive overview of temporal trends in ALL care quality and gender disparities at the global and national levels, rather than to perform inferential statistical comparisons. This point has been clarified in the Methods section.
5. Please declare to what extent have AI chatbots been used in developing your paper and to which parts of the paper did they contribute	We declare that no artificial intelligence (AI) chatbots were used in the development of this paper. All aspects of study design, data analysis, interpretation of results, and manuscript writing were conducted solely by the authors.

Table S2. The mean values of four secondary variables in 1990 and 2021.

	MIR	YLL to YLD	DAL to PER	PER to INC
1990	0.86	159.18	29.86	2.41
2021	0.73	114.58	19.33	3.45

Abbreviations: MIR, mortality-to-incidence ratio; YLL to YLD, years of life lost rate-to-years lived with disability rate tatio; DAL to PER, disability adjusted life years rate-to-prevalence ratio; PER to INC, prevalence-to-incidence ratio.

Table S3. Quality of Care Index (QCI) for acute lymphoblastic leukemia globally and across five SDI regions, 1990-2021.

measure	location	age	cause	year	val	lower	upper
QCI	Global	Age-standardized	Acute lymphoid leukemia	1990	31.35	31.21	31.45
QCI	Global	Age-standardized	Acute lymphoid leukemia	1991	32.00	31.89	32.10
QCI	Global	Age-standardized	Acute lymphoid leukemia	1992	32.79	32.67	32.90
QCI	Global	Age-standardized	Acute lymphoid leukemia	1993	33.58	33.46	33.69
QCI	Global	Age-standardized	Acute lymphoid leukemia	1994	34.63	34.51	34.74
QCI	Global	Age-standardized	Acute lymphoid leukemia	1995	35.83	35.70	35.93
QCI	Global	Age-standardized	Acute lymphoid leukemia	1996	37.19	37.07	37.29
QCI	Global	Age-standardized	Acute lymphoid leukemia	1997	38.20	38.07	38.31
QCI	Global	Age-standardized	Acute lymphoid leukemia	1998	38.66	38.53	38.77
QCI	Global	Age-standardized	Acute lymphoid leukemia	1999	38.98	38.85	39.09
QCI	Global	Age-standardized	Acute lymphoid leukemia	2000	39.60	39.47	39.71
QCI	Global	Age-standardized	Acute lymphoid leukemia	2001	40.19	40.07	40.30
QCI	Global	Age-standardized	Acute lymphoid leukemia	2002	40.99	40.88	41.11
QCI	Global	Age-standardized	Acute lymphoid leukemia	2003	42.08	41.96	42.20
QCI	Global	Age-standardized	Acute lymphoid leukemia	2004	43.14	43.02	43.26
QCI	Global	Age-standardized	Acute lymphoid leukemia	2005	44.87	44.75	44.99
QCI	Global	Age-standardized	Acute lymphoid leukemia	2006	46.97	46.85	47.08
QCI	Global	Age-standardized	Acute lymphoid leukemia	2007	48.53	48.41	48.65
QCI	Global	Age-standardized	Acute lymphoid leukemia	2008	49.80	49.69	49.93
QCI	Global	Age-standardized	Acute lymphoid leukemia	2009	50.74	50.63	50.86
QCI	Global	Age-standardized	Acute lymphoid leukemia	2010	51.73	51.61	51.85
QCI	Global	Age-standardized	Acute lymphoid leukemia	2011	52.56	52.44	52.67
QCI	Global	Age-standardized	Acute lymphoid leukemia	2012	53.67	53.56	53.79
QCI	Global	Age-standardized	Acute lymphoid leukemia	2013	54.74	54.62	54.86
QCI	Global	Age-standardized	Acute lymphoid leukemia	2014	55.99	55.88	56.11
QCI	Global	Age-standardized	Acute lymphoid leukemia	2015	56.76	56.65	56.88
QCI	Global	Age-standardized	Acute lymphoid leukemia	2016	57.32	57.21	57.44
QCI	Global	Age-standardized	Acute lymphoid leukemia	2017	58.82	58.72	58.94
QCI	Global	Age-standardized	Acute lymphoid leukemia	2018	60.31	60.20	60.43
QCI	Global	Age-standardized	Acute lymphoid leukemia	2019	61.16	61.06	61.28
QCI	Global	Age-standardized	Acute lymphoid leukemia	2020	58.66	58.55	58.79
QCI	Global	Age-standardized	Acute lymphoid leukemia	2021	58.46	58.36	58.58
QCI	Low SDI	Age-standardized	Acute lymphoid leukemia	1990	8.01	7.88	8.05
QCI	Low SDI	Age-standardized	Acute lymphoid leukemia	1991	8.03	7.91	8.07
QCI	Low SDI	Age-standardized	Acute lymphoid leukemia	1992	8.04	7.92	8.09
QCI	Low SDI	Age-standardized	Acute lymphoid leukemia	1993	8.11	7.99	8.16
QCI	Low SDI	Age-standardized	Acute lymphoid leukemia	1994	8.17	8.05	8.21
QCI	Low SDI	Age-standardized	Acute lymphoid leukemia	1995	8.38	8.25	8.42
QCI	Low SDI	Age-standardized	Acute lymphoid leukemia	1996	8.58	8.45	8.61
QCI	Low SDI	Age-standardized	Acute lymphoid leukemia	1997	8.76	8.64	8.80
QCI	Low SDI	Age-standardized	Acute lymphoid leukemia	1998	8.94	8.81	8.98
QCI	Low SDI	Age-standardized	Acute lymphoid leukemia	1999	9.00	8.88	9.05
QCI	Low SDI	Age-standardized	Acute lymphoid leukemia	2000	9.01	8.88	9.05
QCI	Low SDI	Age-standardized	Acute lymphoid leukemia	2001	9.06	8.93	9.11
QCI	Low SDI	Age-standardized	Acute lymphoid leukemia	2002	9.33	9.21	9.37
QCI	Low SDI	Age-standardized	Acute lymphoid leukemia	2003	9.50	9.37	9.55
QCI	Low SDI	Age-standardized	Acute lymphoid leukemia	2004	9.49	9.36	9.54
QCI	Low SDI	Age-standardized	Acute lymphoid leukemia	2005	9.47	9.34	9.51
QCI	Low SDI	Age-standardized	Acute lymphoid leukemia	2006	9.59	9.47	9.64
QCI	Low SDI	Age-standardized	Acute lymphoid leukemia	2007	9.77	9.64	9.82
QCI	Low SDI	Age-standardized	Acute lymphoid leukemia	2008	10.07	9.94	10.12
QCI	Low SDI	Age-standardized	Acute lymphoid leukemia	2009	10.39	10.25	10.44
QCI	Low SDI	Age-standardized	Acute lymphoid leukemia	2010	10.70	10.57	10.75
QCI	Low SDI	Age-standardized	Acute lymphoid leukemia	2011	10.90	10.78	10.95
QCI	Low SDI	Age-standardized	Acute lymphoid leukemia	2012	11.00	10.87	11.05
QCI	Low SDI	Age-standardized	Acute lymphoid leukemia	2013	11.10	10.97	11.15
QCI	Low SDI	Age-standardized	Acute lymphoid leukemia	2014	11.32	11.19	11.37
QCI	Low SDI	Age-standardized	Acute lymphoid leukemia	2015	11.71	11.59	11.77
QCI	Low SDI	Age-standardized	Acute lymphoid leukemia	2016	11.98	11.85	12.04
QCI	Low SDI	Age-standardized	Acute lymphoid leukemia	2017	12.25	12.12	12.31
QCI	Low SDI	Age-standardized	Acute lymphoid leukemia	2018	12.67	12.54	12.73
QCI	Low SDI	Age-standardized	Acute lymphoid leukemia	2019	13.09	12.96	13.15
QCI	Low SDI	Age-standardized	Acute lymphoid leukemia	2020	13.59	13.46	13.66
QCI	Low SDI	Age-standardized	Acute lymphoid leukemia	2021	14.10	13.97	14.16
QCI	Low-middle SDI	Age-standardized	Acute lymphoid leukemia	1990	10.35	10.23	10.40
QCI	Low-middle SDI	Age-standardized	Acute lymphoid leukemia	1991	10.66	10.53	10.70
QCI	Low-middle SDI	Age-standardized	Acute lymphoid leukemia	1992	10.90	10.78	10.96

measure	location	age	cause	year	val	lower	upper
QCI	Low-middle SDI	Age-standardized	Acute lymphoid leukemia	1993	11.15	11.02	11.20
QCI	Low-middle SDI	Age-standardized	Acute lymphoid leukemia	1994	11.42	11.29	11.47
QCI	Low-middle SDI	Age-standardized	Acute lymphoid leukemia	1995	11.58	11.45	11.63
QCI	Low-middle SDI	Age-standardized	Acute lymphoid leukemia	1996	11.85	11.72	11.91
QCI	Low-middle SDI	Age-standardized	Acute lymphoid leukemia	1997	12.14	12.01	12.19
QCI	Low-middle SDI	Age-standardized	Acute lymphoid leukemia	1998	12.34	12.21	12.39
QCI	Low-middle SDI	Age-standardized	Acute lymphoid leukemia	1999	12.63	12.50	12.68
QCI	Low-middle SDI	Age-standardized	Acute lymphoid leukemia	2000	12.92	12.79	12.97
QCI	Low-middle SDI	Age-standardized	Acute lymphoid leukemia	2001	13.32	13.21	13.38
QCI	Low-middle SDI	Age-standardized	Acute lymphoid leukemia	2002	13.95	13.82	14.01
QCI	Low-middle SDI	Age-standardized	Acute lymphoid leukemia	2003	14.46	14.33	14.52
QCI	Low-middle SDI	Age-standardized	Acute lymphoid leukemia	2004	14.57	14.45	14.64
QCI	Low-middle SDI	Age-standardized	Acute lymphoid leukemia	2005	14.62	14.49	14.69
QCI	Low-middle SDI	Age-standardized	Acute lymphoid leukemia	2006	15.04	14.91	15.11
QCI	Low-middle SDI	Age-standardized	Acute lymphoid leukemia	2007	15.63	15.50	15.70
QCI	Low-middle SDI	Age-standardized	Acute lymphoid leukemia	2008	16.32	16.19	16.39
QCI	Low-middle SDI	Age-standardized	Acute lymphoid leukemia	2009	17.08	16.96	17.16
QCI	Low-middle SDI	Age-standardized	Acute lymphoid leukemia	2010	17.87	17.74	17.95
QCI	Low-middle SDI	Age-standardized	Acute lymphoid leukemia	2011	18.47	18.34	18.54
QCI	Low-middle SDI	Age-standardized	Acute lymphoid leukemia	2012	18.96	18.83	19.04
QCI	Low-middle SDI	Age-standardized	Acute lymphoid leukemia	2013	19.26	19.14	19.34
QCI	Low-middle SDI	Age-standardized	Acute lymphoid leukemia	2014	19.69	19.57	19.77
QCI	Low-middle SDI	Age-standardized	Acute lymphoid leukemia	2015	20.42	20.28	20.50
QCI	Low-middle SDI	Age-standardized	Acute lymphoid leukemia	2016	21.30	21.17	21.38
QCI	Low-middle SDI	Age-standardized	Acute lymphoid leukemia	2017	21.93	21.80	22.02
QCI	Low-middle SDI	Age-standardized	Acute lymphoid leukemia	2018	22.61	22.47	22.70
QCI	Low-middle SDI	Age-standardized	Acute lymphoid leukemia	2019	23.35	23.22	23.44
QCI	Low-middle SDI	Age-standardized	Acute lymphoid leukemia	2020	23.74	23.60	23.83
QCI	Low-middle SDI	Age-standardized	Acute lymphoid leukemia	2021	24.34	24.22	24.44
QCI	Middle SDI	Age-standardized	Acute lymphoid leukemia	1990	18.28	18.15	18.35
QCI	Middle SDI	Age-standardized	Acute lymphoid leukemia	1991	19.20	19.07	19.27
QCI	Middle SDI	Age-standardized	Acute lymphoid leukemia	1992	20.12	19.99	20.19
QCI	Middle SDI	Age-standardized	Acute lymphoid leukemia	1993	21.10	20.97	21.17
QCI	Middle SDI	Age-standardized	Acute lymphoid leukemia	1994	22.09	21.96	22.17
QCI	Middle SDI	Age-standardized	Acute lymphoid leukemia	1995	23.03	22.90	23.12
QCI	Middle SDI	Age-standardized	Acute lymphoid leukemia	1996	24.10	23.98	24.19
QCI	Middle SDI	Age-standardized	Acute lymphoid leukemia	1997	25.21	25.08	25.30
QCI	Middle SDI	Age-standardized	Acute lymphoid leukemia	1998	26.25	26.12	26.34
QCI	Middle SDI	Age-standardized	Acute lymphoid leukemia	1999	27.36	27.23	27.45
QCI	Middle SDI	Age-standardized	Acute lymphoid leukemia	2000	28.50	28.37	28.59
QCI	Middle SDI	Age-standardized	Acute lymphoid leukemia	2001	29.55	29.42	29.65
QCI	Middle SDI	Age-standardized	Acute lymphoid leukemia	2002	30.69	30.58	30.79
QCI	Middle SDI	Age-standardized	Acute lymphoid leukemia	2003	32.03	31.90	32.14
QCI	Middle SDI	Age-standardized	Acute lymphoid leukemia	2004	33.55	33.42	33.66
QCI	Middle SDI	Age-standardized	Acute lymphoid leukemia	2005	35.55	35.42	35.66
QCI	Middle SDI	Age-standardized	Acute lymphoid leukemia	2006	37.91	37.77	38.02
QCI	Middle SDI	Age-standardized	Acute lymphoid leukemia	2007	39.95	39.83	40.07
QCI	Middle SDI	Age-standardized	Acute lymphoid leukemia	2008	41.77	41.64	41.89
QCI	Middle SDI	Age-standardized	Acute lymphoid leukemia	2009	43.36	43.24	43.48
QCI	Middle SDI	Age-standardized	Acute lymphoid leukemia	2010	45.15	45.02	45.27
QCI	Middle SDI	Age-standardized	Acute lymphoid leukemia	2011	46.91	46.79	47.02
QCI	Middle SDI	Age-standardized	Acute lymphoid leukemia	2012	48.42	48.29	48.53
QCI	Middle SDI	Age-standardized	Acute lymphoid leukemia	2013	50.02	49.90	50.14
QCI	Middle SDI	Age-standardized	Acute lymphoid leukemia	2014	51.91	51.79	52.03
QCI	Middle SDI	Age-standardized	Acute lymphoid leukemia	2015	52.82	52.71	52.94
QCI	Middle SDI	Age-standardized	Acute lymphoid leukemia	2016	53.80	53.68	53.91
QCI	Middle SDI	Age-standardized	Acute lymphoid leukemia	2017	56.25	56.14	56.37
QCI	Middle SDI	Age-standardized	Acute lymphoid leukemia	2018	58.41	58.31	58.53
QCI	Middle SDI	Age-standardized	Acute lymphoid leukemia	2019	59.73	59.62	59.85
QCI	Middle SDI	Age-standardized	Acute lymphoid leukemia	2020	57.44	57.33	57.57
QCI	Middle SDI	Age-standardized	Acute lymphoid leukemia	2021	57.47	57.35	57.59
QCI	High-middle SDI	Age-standardized	Acute lymphoid leukemia	1990	38.11	37.99	38.21
QCI	High-middle SDI	Age-standardized	Acute lymphoid leukemia	1991	39.61	39.50	39.72
QCI	High-middle SDI	Age-standardized	Acute lymphoid leukemia	1992	41.22	41.10	41.33
QCI	High-middle SDI	Age-standardized	Acute lymphoid leukemia	1993	42.66	42.54	42.76
QCI	High-middle SDI	Age-standardized	Acute lymphoid leukemia	1994	44.03	43.92	44.14
QCI	High-middle SDI	Age-standardized	Acute lymphoid leukemia	1995	45.78	45.66	45.88
QCI	High-middle SDI	Age-standardized	Acute lymphoid leukemia	1996	47.89	47.78	48.01

measure	location	age	cause	year	val	lower	upper
QCI	High-middle SDI	Age-standardized	Acute lymphoid leukemia	1997	49.44	49.33	49.54
QCI	High-middle SDI	Age-standardized	Acute lymphoid leukemia	1998	50.43	50.32	50.55
QCI	High-middle SDI	Age-standardized	Acute lymphoid leukemia	1999	51.08	50.96	51.19
QCI	High-middle SDI	Age-standardized	Acute lymphoid leukemia	2000	52.13	52.02	52.24
QCI	High-middle SDI	Age-standardized	Acute lymphoid leukemia	2001	53.32	53.22	53.44
QCI	High-middle SDI	Age-standardized	Acute lymphoid leukemia	2002	54.93	54.82	55.04
QCI	High-middle SDI	Age-standardized	Acute lymphoid leukemia	2003	56.76	56.65	56.87
QCI	High-middle SDI	Age-standardized	Acute lymphoid leukemia	2004	59.41	59.31	59.53
QCI	High-middle SDI	Age-standardized	Acute lymphoid leukemia	2005	62.98	62.88	63.10
QCI	High-middle SDI	Age-standardized	Acute lymphoid leukemia	2006	66.77	66.68	66.89
QCI	High-middle SDI	Age-standardized	Acute lymphoid leukemia	2007	69.08	68.99	69.18
QCI	High-middle SDI	Age-standardized	Acute lymphoid leukemia	2008	70.69	70.60	70.80
QCI	High-middle SDI	Age-standardized	Acute lymphoid leukemia	2009	71.87	71.78	71.98
QCI	High-middle SDI	Age-standardized	Acute lymphoid leukemia	2010	73.24	73.16	73.34
QCI	High-middle SDI	Age-standardized	Acute lymphoid leukemia	2011	74.88	74.81	75.00
QCI	High-middle SDI	Age-standardized	Acute lymphoid leukemia	2012	76.51	76.44	76.62
QCI	High-middle SDI	Age-standardized	Acute lymphoid leukemia	2013	78.11	78.04	78.22
QCI	High-middle SDI	Age-standardized	Acute lymphoid leukemia	2014	79.65	79.58	79.75
QCI	High-middle SDI	Age-standardized	Acute lymphoid leukemia	2015	80.33	80.26	80.43
QCI	High-middle SDI	Age-standardized	Acute lymphoid leukemia	2016	80.85	80.79	80.96
QCI	High-middle SDI	Age-standardized	Acute lymphoid leukemia	2017	82.39	82.34	82.49
QCI	High-middle SDI	Age-standardized	Acute lymphoid leukemia	2018	83.82	83.76	83.92
QCI	High-middle SDI	Age-standardized	Acute lymphoid leukemia	2019	84.55	84.51	84.65
QCI	High-middle SDI	Age-standardized	Acute lymphoid leukemia	2020	82.73	82.67	82.83
QCI	High-middle SDI	Age-standardized	Acute lymphoid leukemia	2021	82.68	82.62	82.77
QCI	High SDI	Age-standardized	Acute lymphoid leukemia	1990	75.82	75.75	75.93
QCI	High SDI	Age-standardized	Acute lymphoid leukemia	1991	76.26	76.19	76.36
QCI	High SDI	Age-standardized	Acute lymphoid leukemia	1992	77.15	77.07	77.25
QCI	High SDI	Age-standardized	Acute lymphoid leukemia	1993	78.05	77.98	78.15
QCI	High SDI	Age-standardized	Acute lymphoid leukemia	1994	79.51	79.45	79.62
QCI	High SDI	Age-standardized	Acute lymphoid leukemia	1995	81.17	81.10	81.26
QCI	High SDI	Age-standardized	Acute lymphoid leukemia	1996	82.66	82.61	82.75
QCI	High SDI	Age-standardized	Acute lymphoid leukemia	1997	83.62	83.56	83.71
QCI	High SDI	Age-standardized	Acute lymphoid leukemia	1998	83.79	83.74	83.88
QCI	High SDI	Age-standardized	Acute lymphoid leukemia	1999	83.88	83.82	83.99
QCI	High SDI	Age-standardized	Acute lymphoid leukemia	2000	84.46	84.41	84.58
QCI	High SDI	Age-standardized	Acute lymphoid leukemia	2001	84.72	84.67	84.81
QCI	High SDI	Age-standardized	Acute lymphoid leukemia	2002	85.17	85.12	85.26
QCI	High SDI	Age-standardized	Acute lymphoid leukemia	2003	86.05	86.01	86.14
QCI	High SDI	Age-standardized	Acute lymphoid leukemia	2004	86.65	86.61	86.74
QCI	High SDI	Age-standardized	Acute lymphoid leukemia	2005	87.50	87.46	87.60
QCI	High SDI	Age-standardized	Acute lymphoid leukemia	2006	88.45	88.41	88.53
QCI	High SDI	Age-standardized	Acute lymphoid leukemia	2007	89.04	89.00	89.14
QCI	High SDI	Age-standardized	Acute lymphoid leukemia	2008	89.63	89.60	89.73
QCI	High SDI	Age-standardized	Acute lymphoid leukemia	2009	89.69	89.65	89.78
QCI	High SDI	Age-standardized	Acute lymphoid leukemia	2010	89.79	89.76	89.88
QCI	High SDI	Age-standardized	Acute lymphoid leukemia	2011	89.77	89.73	89.87
QCI	High SDI	Age-standardized	Acute lymphoid leukemia	2012	90.31	90.28	90.41
QCI	High SDI	Age-standardized	Acute lymphoid leukemia	2013	90.37	90.34	90.47
QCI	High SDI	Age-standardized	Acute lymphoid leukemia	2014	90.54	90.51	90.63
QCI	High SDI	Age-standardized	Acute lymphoid leukemia	2015	90.95	90.92	91.05
QCI	High SDI	Age-standardized	Acute lymphoid leukemia	2016	90.88	90.85	90.98
QCI	High SDI	Age-standardized	Acute lymphoid leukemia	2017	91.03	91.00	91.13
QCI	High SDI	Age-standardized	Acute lymphoid leukemia	2018	91.17	91.14	91.27
QCI	High SDI	Age-standardized	Acute lymphoid leukemia	2019	91.72	91.69	91.81
QCI	High SDI	Age-standardized	Acute lymphoid leukemia	2020	90.92	90.89	91.02
QCI	High SDI	Age-standardized	Acute lymphoid leukemia	2021	90.75	90.72	90.85

Table S4. Gender disparity ratio (GDR) of Quality of Care Index (QCI) for acute lymphoblastic leukemia globally and across five SDI regions, 1990-2021.

measure	location	age	cause	year	val
GDR	Global	Age-standardized	Acute lymphoid leukemia	1990	0.96
GDR	Global	Age-standardized	Acute lymphoid leukemia	1991	0.97
GDR	Global	Age-standardized	Acute lymphoid leukemia	1992	0.96
GDR	Global	Age-standardized	Acute lymphoid leukemia	1993	0.97
GDR	Global	Age-standardized	Acute lymphoid leukemia	1994	0.97
GDR	Global	Age-standardized	Acute lymphoid leukemia	1995	0.97
GDR	Global	Age-standardized	Acute lymphoid leukemia	1996	0.98
GDR	Global	Age-standardized	Acute lymphoid leukemia	1997	0.99
GDR	Global	Age-standardized	Acute lymphoid leukemia	1998	0.99
GDR	Global	Age-standardized	Acute lymphoid leukemia	1999	0.98
GDR	Global	Age-standardized	Acute lymphoid leukemia	2000	0.99
GDR	Global	Age-standardized	Acute lymphoid leukemia	2001	0.99
GDR	Global	Age-standardized	Acute lymphoid leukemia	2002	1.00
GDR	Global	Age-standardized	Acute lymphoid leukemia	2003	0.99
GDR	Global	Age-standardized	Acute lymphoid leukemia	2004	1.00
GDR	Global	Age-standardized	Acute lymphoid leukemia	2005	0.99
GDR	Global	Age-standardized	Acute lymphoid leukemia	2006	0.99
GDR	Global	Age-standardized	Acute lymphoid leukemia	2007	1.00
GDR	Global	Age-standardized	Acute lymphoid leukemia	2008	1.00
GDR	Global	Age-standardized	Acute lymphoid leukemia	2009	1.01
GDR	Global	Age-standardized	Acute lymphoid leukemia	2010	1.02
GDR	Global	Age-standardized	Acute lymphoid leukemia	2011	1.02
GDR	Global	Age-standardized	Acute lymphoid leukemia	2012	1.02
GDR	Global	Age-standardized	Acute lymphoid leukemia	2013	1.02
GDR	Global	Age-standardized	Acute lymphoid leukemia	2014	1.02
GDR	Global	Age-standardized	Acute lymphoid leukemia	2015	1.02
GDR	Global	Age-standardized	Acute lymphoid leukemia	2016	1.02
GDR	Global	Age-standardized	Acute lymphoid leukemia	2017	1.01
GDR	Global	Age-standardized	Acute lymphoid leukemia	2018	1.02
GDR	Global	Age-standardized	Acute lymphoid leukemia	2019	1.01
GDR	Global	Age-standardized	Acute lymphoid leukemia	2020	1.01
GDR	Global	Age-standardized	Acute lymphoid leukemia	2021	1.00
GDR	Low SDI	Age-standardized	Acute lymphoid leukemia	1990	1.02
GDR	Low SDI	Age-standardized	Acute lymphoid leukemia	1991	1.01
GDR	Low SDI	Age-standardized	Acute lymphoid leukemia	1992	1.00
GDR	Low SDI	Age-standardized	Acute lymphoid leukemia	1993	0.99
GDR	Low SDI	Age-standardized	Acute lymphoid leukemia	1994	1.00
GDR	Low SDI	Age-standardized	Acute lymphoid leukemia	1995	0.99
GDR	Low SDI	Age-standardized	Acute lymphoid leukemia	1996	0.98
GDR	Low SDI	Age-standardized	Acute lymphoid leukemia	1997	0.96
GDR	Low SDI	Age-standardized	Acute lymphoid leukemia	1998	0.96
GDR	Low SDI	Age-standardized	Acute lymphoid leukemia	1999	0.95
GDR	Low SDI	Age-standardized	Acute lymphoid leukemia	2000	0.95
GDR	Low SDI	Age-standardized	Acute lymphoid leukemia	2001	0.96
GDR	Low SDI	Age-standardized	Acute lymphoid leukemia	2002	0.95
GDR	Low SDI	Age-standardized	Acute lymphoid leukemia	2003	0.94
GDR	Low SDI	Age-standardized	Acute lymphoid leukemia	2004	0.93
GDR	Low SDI	Age-standardized	Acute lymphoid leukemia	2005	0.92
GDR	Low SDI	Age-standardized	Acute lymphoid leukemia	2006	0.92
GDR	Low SDI	Age-standardized	Acute lymphoid leukemia	2007	0.92
GDR	Low SDI	Age-standardized	Acute lymphoid leukemia	2008	0.93
GDR	Low SDI	Age-standardized	Acute lymphoid leukemia	2009	0.93
GDR	Low SDI	Age-standardized	Acute lymphoid leukemia	2010	0.94
GDR	Low SDI	Age-standardized	Acute lymphoid leukemia	2011	0.94
GDR	Low SDI	Age-standardized	Acute lymphoid leukemia	2012	0.95
GDR	Low SDI	Age-standardized	Acute lymphoid leukemia	2013	0.95
GDR	Low SDI	Age-standardized	Acute lymphoid leukemia	2014	0.95
GDR	Low SDI	Age-standardized	Acute lymphoid leukemia	2015	0.95
GDR	Low SDI	Age-standardized	Acute lymphoid leukemia	2016	0.95
GDR	Low SDI	Age-standardized	Acute lymphoid leukemia	2017	0.94
GDR	Low SDI	Age-standardized	Acute lymphoid leukemia	2018	0.94
GDR	Low SDI	Age-standardized	Acute lymphoid leukemia	2019	0.94
GDR	Low SDI	Age-standardized	Acute lymphoid leukemia	2020	0.93
GDR	Low SDI	Age-standardized	Acute lymphoid leukemia	2021	0.93
GDR	Low-middle SDI	Age-standardized	Acute lymphoid leukemia	1990	0.76
GDR	Low-middle SDI	Age-standardized	Acute lymphoid leukemia	1991	0.76
GDR	Low-middle SDI	Age-standardized	Acute lymphoid leukemia	1992	0.77
GDR	Low-middle SDI	Age-standardized	Acute lymphoid leukemia	1993	0.77
GDR	Low-middle SDI	Age-standardized	Acute lymphoid leukemia	1994	0.78
GDR	Low-middle SDI	Age-standardized	Acute lymphoid leukemia	1995	0.78
GDR	Low-middle SDI	Age-standardized	Acute lymphoid leukemia	1996	0.79
GDR	Low-middle SDI	Age-standardized	Acute lymphoid leukemia	1997	0.80
GDR	Low-middle SDI	Age-standardized	Acute lymphoid leukemia	1998	0.81
GDR	Low-middle SDI	Age-standardized	Acute lymphoid leukemia	1999	0.80
GDR	Low-middle SDI	Age-standardized	Acute lymphoid leukemia	2000	0.81
GDR	Low-middle SDI	Age-standardized	Acute lymphoid leukemia	2001	0.83
GDR	Low-middle SDI	Age-standardized	Acute lymphoid leukemia	2002	0.84
GDR	Low-middle SDI	Age-standardized	Acute lymphoid leukemia	2003	0.85
GDR	Low-middle SDI	Age-standardized	Acute lymphoid leukemia	2004	0.86
GDR	Low-middle SDI	Age-standardized	Acute lymphoid leukemia	2005	0.87
GDR	Low-middle SDI	Age-standardized	Acute lymphoid leukemia	2006	0.88
GDR	Low-middle SDI	Age-standardized	Acute lymphoid leukemia	2007	0.90
GDR	Low-middle SDI	Age-standardized	Acute lymphoid leukemia	2008	0.90
GDR	Low-middle SDI	Age-standardized	Acute lymphoid leukemia	2009	0.91
GDR	Low-middle SDI	Age-standardized	Acute lymphoid leukemia	2010	0.94
GDR	Low-middle SDI	Age-standardized	Acute lymphoid leukemia	2011	0.94
GDR	Low-middle SDI	Age-standardized	Acute lymphoid leukemia	2012	0.96

measure	location	age	cause	year	val
GDR	Low-middle SDI	Age-standardized	Acute lymphoid leukemia	2013	0.95
GDR	Low-middle SDI	Age-standardized	Acute lymphoid leukemia	2014	0.96
GDR	Low-middle SDI	Age-standardized	Acute lymphoid leukemia	2015	0.97
GDR	Low-middle SDI	Age-standardized	Acute lymphoid leukemia	2016	0.97
GDR	Low-middle SDI	Age-standardized	Acute lymphoid leukemia	2017	0.97
GDR	Low-middle SDI	Age-standardized	Acute lymphoid leukemia	2018	0.98
GDR	Low-middle SDI	Age-standardized	Acute lymphoid leukemia	2019	0.99
GDR	Low-middle SDI	Age-standardized	Acute lymphoid leukemia	2020	0.97
GDR	Low-middle SDI	Age-standardized	Acute lymphoid leukemia	2021	0.97
GDR	Middle SDI	Age-standardized	Acute lymphoid leukemia	1990	0.99
GDR	Middle SDI	Age-standardized	Acute lymphoid leukemia	1991	1.00
GDR	Middle SDI	Age-standardized	Acute lymphoid leukemia	1992	1.00
GDR	Middle SDI	Age-standardized	Acute lymphoid leukemia	1993	1.01
GDR	Middle SDI	Age-standardized	Acute lymphoid leukemia	1994	1.02
GDR	Middle SDI	Age-standardized	Acute lymphoid leukemia	1995	1.03
GDR	Middle SDI	Age-standardized	Acute lymphoid leukemia	1996	1.03
GDR	Middle SDI	Age-standardized	Acute lymphoid leukemia	1997	1.03
GDR	Middle SDI	Age-standardized	Acute lymphoid leukemia	1998	1.04
GDR	Middle SDI	Age-standardized	Acute lymphoid leukemia	1999	1.03
GDR	Middle SDI	Age-standardized	Acute lymphoid leukemia	2000	1.03
GDR	Middle SDI	Age-standardized	Acute lymphoid leukemia	2001	1.04
GDR	Middle SDI	Age-standardized	Acute lymphoid leukemia	2002	1.03
GDR	Middle SDI	Age-standardized	Acute lymphoid leukemia	2003	1.03
GDR	Middle SDI	Age-standardized	Acute lymphoid leukemia	2004	1.03
GDR	Middle SDI	Age-standardized	Acute lymphoid leukemia	2005	1.02
GDR	Middle SDI	Age-standardized	Acute lymphoid leukemia	2006	1.02
GDR	Middle SDI	Age-standardized	Acute lymphoid leukemia	2007	1.02
GDR	Middle SDI	Age-standardized	Acute lymphoid leukemia	2008	1.02
GDR	Middle SDI	Age-standardized	Acute lymphoid leukemia	2009	1.02
GDR	Middle SDI	Age-standardized	Acute lymphoid leukemia	2010	1.03
GDR	Middle SDI	Age-standardized	Acute lymphoid leukemia	2011	1.03
GDR	Middle SDI	Age-standardized	Acute lymphoid leukemia	2012	1.03
GDR	Middle SDI	Age-standardized	Acute lymphoid leukemia	2013	1.04
GDR	Middle SDI	Age-standardized	Acute lymphoid leukemia	2014	1.04
GDR	Middle SDI	Age-standardized	Acute lymphoid leukemia	2015	1.04
GDR	Middle SDI	Age-standardized	Acute lymphoid leukemia	2016	1.04
GDR	Middle SDI	Age-standardized	Acute lymphoid leukemia	2017	1.03
GDR	Middle SDI	Age-standardized	Acute lymphoid leukemia	2018	1.03
GDR	Middle SDI	Age-standardized	Acute lymphoid leukemia	2019	1.02
GDR	Middle SDI	Age-standardized	Acute lymphoid leukemia	2020	1.01
GDR	Middle SDI	Age-standardized	Acute lymphoid leukemia	2021	1.01
GDR	High-middle SDI	Age-standardized	Acute lymphoid leukemia	1990	0.99
GDR	High-middle SDI	Age-standardized	Acute lymphoid leukemia	1991	0.99
GDR	High-middle SDI	Age-standardized	Acute lymphoid leukemia	1992	1.00
GDR	High-middle SDI	Age-standardized	Acute lymphoid leukemia	1993	1.00
GDR	High-middle SDI	Age-standardized	Acute lymphoid leukemia	1994	1.01
GDR	High-middle SDI	Age-standardized	Acute lymphoid leukemia	1995	1.01
GDR	High-middle SDI	Age-standardized	Acute lymphoid leukemia	1996	1.01
GDR	High-middle SDI	Age-standardized	Acute lymphoid leukemia	1997	1.01
GDR	High-middle SDI	Age-standardized	Acute lymphoid leukemia	1998	1.02
GDR	High-middle SDI	Age-standardized	Acute lymphoid leukemia	1999	1.02
GDR	High-middle SDI	Age-standardized	Acute lymphoid leukemia	2000	1.02
GDR	High-middle SDI	Age-standardized	Acute lymphoid leukemia	2001	1.02
GDR	High-middle SDI	Age-standardized	Acute lymphoid leukemia	2002	1.02
GDR	High-middle SDI	Age-standardized	Acute lymphoid leukemia	2003	1.03
GDR	High-middle SDI	Age-standardized	Acute lymphoid leukemia	2004	1.03
GDR	High-middle SDI	Age-standardized	Acute lymphoid leukemia	2005	1.02
GDR	High-middle SDI	Age-standardized	Acute lymphoid leukemia	2006	1.02
GDR	High-middle SDI	Age-standardized	Acute lymphoid leukemia	2007	1.02
GDR	High-middle SDI	Age-standardized	Acute lymphoid leukemia	2008	1.02
GDR	High-middle SDI	Age-standardized	Acute lymphoid leukemia	2009	1.03
GDR	High-middle SDI	Age-standardized	Acute lymphoid leukemia	2010	1.03
GDR	High-middle SDI	Age-standardized	Acute lymphoid leukemia	2011	1.03
GDR	High-middle SDI	Age-standardized	Acute lymphoid leukemia	2012	1.03
GDR	High-middle SDI	Age-standardized	Acute lymphoid leukemia	2013	1.03
GDR	High-middle SDI	Age-standardized	Acute lymphoid leukemia	2014	1.03
GDR	High-middle SDI	Age-standardized	Acute lymphoid leukemia	2015	1.02
GDR	High-middle SDI	Age-standardized	Acute lymphoid leukemia	2016	1.02
GDR	High-middle SDI	Age-standardized	Acute lymphoid leukemia	2017	1.02
GDR	High-middle SDI	Age-standardized	Acute lymphoid leukemia	2018	1.02
GDR	High-middle SDI	Age-standardized	Acute lymphoid leukemia	2019	1.02
GDR	High-middle SDI	Age-standardized	Acute lymphoid leukemia	2020	1.01
GDR	High-middle SDI	Age-standardized	Acute lymphoid leukemia	2021	1.01
GDR	High SDI	Age-standardized	Acute lymphoid leukemia	1990	1.02
GDR	High SDI	Age-standardized	Acute lymphoid leukemia	1991	1.02
GDR	High SDI	Age-standardized	Acute lymphoid leukemia	1992	1.01
GDR	High SDI	Age-standardized	Acute lymphoid leukemia	1993	1.01
GDR	High SDI	Age-standardized	Acute lymphoid leukemia	1994	1.02
GDR	High SDI	Age-standardized	Acute lymphoid leukemia	1995	1.01
GDR	High SDI	Age-standardized	Acute lymphoid leukemia	1996	1.01
GDR	High SDI	Age-standardized	Acute lymphoid leukemia	1997	1.02
GDR	High SDI	Age-standardized	Acute lymphoid leukemia	1998	1.01
GDR	High SDI	Age-standardized	Acute lymphoid leukemia	1999	1.01
GDR	High SDI	Age-standardized	Acute lymphoid leukemia	2000	1.01
GDR	High SDI	Age-standardized	Acute lymphoid leukemia	2001	1.01
GDR	High SDI	Age-standardized	Acute lymphoid leukemia	2002	1.01
GDR	High SDI	Age-standardized	Acute lymphoid leukemia	2003	1.00
GDR	High SDI	Age-standardized	Acute lymphoid leukemia	2004	1.00

measure	location	age	cause	year	val
GDR	High SDI	Age-standardized	Acute lymphoid leukemia	2005	0.99
GDR	High SDI	Age-standardized	Acute lymphoid leukemia	2006	1.00
GDR	High SDI	Age-standardized	Acute lymphoid leukemia	2007	1.00
GDR	High SDI	Age-standardized	Acute lymphoid leukemia	2008	1.00
GDR	High SDI	Age-standardized	Acute lymphoid leukemia	2009	1.01
GDR	High SDI	Age-standardized	Acute lymphoid leukemia	2010	1.01
GDR	High SDI	Age-standardized	Acute lymphoid leukemia	2011	1.01
GDR	High SDI	Age-standardized	Acute lymphoid leukemia	2012	1.01
GDR	High SDI	Age-standardized	Acute lymphoid leukemia	2013	1.01
GDR	High SDI	Age-standardized	Acute lymphoid leukemia	2014	1.01
GDR	High SDI	Age-standardized	Acute lymphoid leukemia	2015	1.01
GDR	High SDI	Age-standardized	Acute lymphoid leukemia	2016	1.00
GDR	High SDI	Age-standardized	Acute lymphoid leukemia	2017	1.00
GDR	High SDI	Age-standardized	Acute lymphoid leukemia	2018	1.01
GDR	High SDI	Age-standardized	Acute lymphoid leukemia	2019	1.01
GDR	High SDI	Age-standardized	Acute lymphoid leukemia	2020	1.01
GDR	High SDI	Age-standardized	Acute lymphoid leukemia	2021	1.00

Table S5. Quality of Care Index (QCI) for acute lymphoblastic leukemia in 204 countries and territories in 1990 and 2021.

measure	group	location	age	cause	year	val	lower	upper
QCI	**Global**	**Global**	Age-standardized	Acute lymphoid leukemia	1990	31.35	31.21	31.45
QCI	**SDI**	**Low SDI**	Age-standardized	Acute lymphoid leukemia	1990	8.01	7.88	8.05
QCI	**SDI**	**Low-middle SDI**	Age-standardized	Acute lymphoid leukemia	1990	10.35	10.23	10.40
QCI	**SDI**	**Middle SDI**	Age-standardized	Acute lymphoid leukemia	1990	18.28	18.15	18.35
QCI	**SDI**	**High-middle SDI**	Age-standardized	Acute lymphoid leukemia	1990	38.11	37.99	38.21
QCI	**SDI**	**High SDI**	Age-standardized	Acute lymphoid leukemia	1990	75.82	75.75	75.93
QCI	**region**	**High-income North America**	Age-standardized	Acute lymphoid leukemia	1990	81.01	80.95	81.12
QCI		Canada	Age-standardized	Acute lymphoid leukemia	1990	86.87	86.82	86.96
QCI		Greenland	Age-standardized	Acute lymphoid leukemia	1990	25.74	25.60	25.85
QCI		United States of America	Age-standardized	Acute lymphoid leukemia	1990	80.28	80.22	80.38
QCI	**region**	**Australasia**	Age-standardized	Acute lymphoid leukemia	1990	69.04	68.96	69.16
QCI		Australia	Age-standardized	Acute lymphoid leukemia	1990	69.43	69.34	69.54
QCI		New Zealand	Age-standardized	Acute lymphoid leukemia	1990	67.14	67.05	67.25
QCI	**region**	**High-income Asia Pacific**	Age-standardized	Acute lymphoid leukemia	1990	72.67	72.59	72.77
QCI		Brunei Darussalam	Age-standardized	Acute lymphoid leukemia	1990	22.51	22.37	22.60
QCI		Japan	Age-standardized	Acute lymphoid leukemia	1990	82.54	82.49	82.63
QCI		Republic of Korea	Age-standardized	Acute lymphoid leukemia	1990	32.53	32.40	32.63
QCI		Singapore	Age-standardized	Acute lymphoid leukemia	1990	55.69	55.57	55.80
QCI	**region**	**Western Europe**	Age-standardized	Acute lymphoid leukemia	1990	84.25	84.20	84.34
QCI		Andorra	Age-standardized	Acute lymphoid leukemia	1990	92.70	92.68	92.80
QCI		Austria	Age-standardized	Acute lymphoid leukemia	1990	84.24	84.19	84.34
QCI		Belgium	Age-standardized	Acute lymphoid leukemia	1990	85.03	84.98	85.12
QCI		Cyprus	Age-standardized	Acute lymphoid leukemia	1990	58.38	58.25	58.52
QCI		Denmark	Age-standardized	Acute lymphoid leukemia	1990	75.59	75.51	75.69
QCI		Finland	Age-standardized	Acute lymphoid leukemia	1990	65.79	65.68	65.91
QCI		France	Age-standardized	Acute lymphoid leukemia	1990	79.18	79.11	79.28
QCI		Germany	Age-standardized	Acute lymphoid leukemia	1990	77.40	77.33	77.50
QCI		Greece	Age-standardized	Acute lymphoid leukemia	1990	89.22	89.19	89.32
QCI		Iceland	Age-standardized	Acute lymphoid leukemia	1990	88.96	88.92	89.05
QCI		Ireland	Age-standardized	Acute lymphoid leukemia	1990	78.27	78.21	78.39
QCI		Israel	Age-standardized	Acute lymphoid leukemia	1990	75.27	75.20	75.38
QCI		Italy	Age-standardized	Acute lymphoid leukemia	1990	89.25	89.22	89.34
QCI		Luxembourg	Age-standardized	Acute lymphoid leukemia	1990	77.17	77.10	77.26
QCI		Malta	Age-standardized	Acute lymphoid leukemia	1990	75.23	75.16	75.35
QCI		Monaco	Age-standardized	Acute lymphoid leukemia	1990	97.30	97.29	97.40
QCI		Netherlands	Age-standardized	Acute lymphoid leukemia	1990	89.57	89.54	89.66
QCI		Norway	Age-standardized	Acute lymphoid leukemia	1990	82.84	82.79	82.95
QCI		Portugal	Age-standardized	Acute lymphoid leukemia	1990	62.40	62.30	62.51
QCI		San Marino	Age-standardized	Acute lymphoid leukemia	1990	96.41	96.40	96.51
QCI		Spain	Age-standardized	Acute lymphoid leukemia	1990	82.83	82.78	82.92
QCI		Sweden	Age-standardized	Acute lymphoid leukemia	1990	93.28	93.25	93.37
QCI		Switzerland	Age-standardized	Acute lymphoid leukemia	1990	94.39	94.37	94.48
QCI		United Kingdom	Age-standardized	Acute lymphoid leukemia	1990	88.95	88.92	89.04
QCI	**region**	**Southern Latin America**	Age-standardized	Acute lymphoid leukemia	1990	27.88	27.74	27.97
QCI		Argentina	Age-standardized	Acute lymphoid leukemia	1990	26.54	26.41	26.63
QCI		Chile	Age-standardized	Acute lymphoid leukemia	1990	29.80	29.66	29.91
QCI		Uruguay	Age-standardized	Acute lymphoid leukemia	1990	35.89	35.77	35.99
QCI	**region**	**Eastern Europe**	Age-standardized	Acute lymphoid leukemia	1990	40.76	40.64	40.87
QCI		Belarus	Age-standardized	Acute lymphoid leukemia	1990	40.24	40.12	40.35
QCI		Estonia	Age-standardized	Acute lymphoid leukemia	1990	46.00	45.89	46.11
QCI		Latvia	Age-standardized	Acute lymphoid leukemia	1990	42.96	42.83	43.08
QCI		Lithuania	Age-standardized	Acute lymphoid leukemia	1990	49.12	49.00	49.22
QCI		Republic of Moldova	Age-standardized	Acute lymphoid leukemia	1990	32.19	32.06	32.30
QCI		Russian Federation	Age-standardized	Acute lymphoid leukemia	1990	38.70	38.58	38.81
QCI		Ukraine	Age-standardized	Acute lymphoid leukemia	1990	44.99	44.87	45.10
QCI	**region**	**Central Europe**	Age-standardized	Acute lymphoid leukemia	1990	32.96	32.83	33.07
QCI		Albania	Age-standardized	Acute lymphoid leukemia	1990	22.53	22.39	22.61
QCI		Bosnia and Herzegovina	Age-standardized	Acute lymphoid leukemia	1990	27.43	27.30	27.54
QCI		Bulgaria	Age-standardized	Acute lymphoid leukemia	1990	34.80	34.67	34.90
QCI		Croatia	Age-standardized	Acute lymphoid leukemia	1990	46.87	46.72	47.01
QCI		Czechia	Age-standardized	Acute lymphoid leukemia	1990	41.57	41.43	41.72
QCI		Hungary	Age-standardized	Acute lymphoid leukemia	1990	36.13	36.00	36.25
QCI		Montenegro	Age-standardized	Acute lymphoid leukemia	1990	62.31	62.21	62.43
QCI		North Macedonia	Age-standardized	Acute lymphoid leukemia	1990	29.06	28.93	29.17
QCI		Poland	Age-standardized	Acute lymphoid leukemia	1990	30.26	30.13	30.36
QCI		Romania	Age-standardized	Acute lymphoid leukemia	1990	26.04	25.91	26.12
QCI		Serbia	Age-standardized	Acute lymphoid leukemia	1990	33.24	33.11	33.34
QCI		Slovakia	Age-standardized	Acute lymphoid leukemia	1990	36.27	36.13	36.38
QCI		Slovenia	Age-standardized	Acute lymphoid leukemia	1990	52.36	52.24	52.48
QCI	**region**	**Central Asia**	Age-standardized	Acute lymphoid leukemia	1990	19.52	19.40	19.59
QCI		Armenia	Age-standardized	Acute lymphoid leukemia	1990	28.83	28.71	28.93
QCI		Azerbaijan	Age-standardized	Acute lymphoid leukemia	1990	16.11	15.99	16.17
QCI		Georgia	Age-standardized	Acute lymphoid leukemia	1990	31.37	31.25	31.46
QCI		Kazakhstan	Age-standardized	Acute lymphoid leukemia	1990	22.11	21.97	22.19
QCI		Kyrgyzstan	Age-standardized	Acute lymphoid leukemia	1990	15.93	15.82	15.99
QCI		Mongolia	Age-standardized	Acute lymphoid leukemia	1990	10.43	10.30	10.47
QCI		Tajikistan	Age-standardized	Acute lymphoid leukemia	1990	13.92	13.80	13.97
QCI		Turkmenistan	Age-standardized	Acute lymphoid leukemia	1990	14.14	14.02	14.20

measure	group	location	age	cause	year	val	lower	upper
QCI		Uzbekistan	Age-standardized	Acute lymphoid leukemia	1990	18.57	18.45	18.64
QCI	region	**Central Latin America**	Age-standardized	Acute lymphoid leukemia	1990	16.37	16.25	16.43
QCI		Colombia	Age-standardized	Acute lymphoid leukemia	1990	17.34	17.22	17.41
QCI		Costa Rica	Age-standardized	Acute lymphoid leukemia	1990	38.00	37.87	38.12
QCI		El Salvador	Age-standardized	Acute lymphoid leukemia	1990	12.48	12.36	12.53
QCI		Guatemala	Age-standardized	Acute lymphoid leukemia	1990	7.28	7.15	7.32
QCI		Honduras	Age-standardized	Acute lymphoid leukemia	1990	10.10	9.98	10.15
QCI		Mexico	Age-standardized	Acute lymphoid leukemia	1990	16.54	16.41	16.60
QCI		Nicaragua	Age-standardized	Acute lymphoid leukemia	1990	16.48	16.37	16.54
QCI		Panama	Age-standardized	Acute lymphoid leukemia	1990	26.35	26.23	26.44
QCI		Venezuela (Bolivarian Republic of)	Age-standardized	Acute lymphoid leukemia	1990	16.02	15.89	16.08
QCI	region	**Andean Latin America**	Age-standardized	Acute lymphoid leukemia	1990	11.75	11.62	11.80
QCI		Bolivia (Plurinational State of)	Age-standardized	Acute lymphoid leukemia	1990	7.92	7.79	7.96
QCI		Ecuador	Age-standardized	Acute lymphoid leukemia	1990	13.34	13.21	13.40
QCI		Peru	Age-standardized	Acute lymphoid leukemia	1990	13.19	13.06	13.24
QCI	region	**Caribbean**	Age-standardized	Acute lymphoid leukemia	1990	20.41	20.28	20.48
QCI		Antigua and Barbuda	Age-standardized	Acute lymphoid leukemia	1990	29.66	29.53	29.76
QCI		Bahamas	Age-standardized	Acute lymphoid leukemia	1990	22.16	22.03	22.23
QCI		Barbados	Age-standardized	Acute lymphoid leukemia	1990	29.71	29.58	29.80
QCI		Bermuda	Age-standardized	Acute lymphoid leukemia	1990	52.10	51.96	52.23
QCI		Belize	Age-standardized	Acute lymphoid leukemia	1990	20.87	20.74	20.93
QCI		Cuba	Age-standardized	Acute lymphoid leukemia	1990	41.82	41.70	41.93
QCI		Dominica	Age-standardized	Acute lymphoid leukemia	1990	19.13	19.00	19.22
QCI		Dominican Republic	Age-standardized	Acute lymphoid leukemia	1990	11.87	11.75	11.91
QCI		Grenada	Age-standardized	Acute lymphoid leukemia	1990	13.58	13.46	13.62
QCI		Guyana	Age-standardized	Acute lymphoid leukemia	1990	8.42	8.30	8.46
QCI		Haiti	Age-standardized	Acute lymphoid leukemia	1990	2.53	2.42	2.55
QCI		Jamaica	Age-standardized	Acute lymphoid leukemia	1990	34.05	33.94	34.14
QCI		Puerto Rico	Age-standardized	Acute lymphoid leukemia	1990	41.84	41.71	41.95
QCI		Saint Kitts and Nevis	Age-standardized	Acute lymphoid leukemia	1990	10.28	10.16	10.32
QCI		Saint Lucia	Age-standardized	Acute lymphoid leukemia	1990	17.72	17.59	17.79
QCI		Saint Vincent and the Grenadines	Age-standardized	Acute lymphoid leukemia	1990	15.58	15.46	15.63
QCI		Suriname	Age-standardized	Acute lymphoid leukemia	1990	10.24	10.12	10.28
QCI		Trinidad and Tobago	Age-standardized	Acute lymphoid leukemia	1990	16.70	16.57	16.77
QCI		United States Virgin Islands	Age-standardized	Acute lymphoid leukemia	1990	26.52	26.39	26.60
QCI	region	**Tropical Latin America**	Age-standardized	Acute lymphoid leukemia	1990	16.70	16.56	16.76
QCI		Brazil	Age-standardized	Acute lymphoid leukemia	1990	16.51	16.39	16.58
QCI		Paraguay	Age-standardized	Acute lymphoid leukemia	1990	20.18	20.05	20.25
QCI	region	**East Asia**	Age-standardized	Acute lymphoid leukemia	1990	23.11	22.98	23.19
QCI		China	Age-standardized	Acute lymphoid leukemia	1990	23.01	22.88	23.09
QCI		Democratic People's Republic of Korea	Age-standardized	Acute lymphoid leukemia	1990	22.25	22.11	22.33
QCI		Taiwan (Province of China)	Age-standardized	Acute lymphoid leukemia	1990	49.40	49.28	49.52
QCI	region	**Southeast Asia**	Age-standardized	Acute lymphoid leukemia	1990	14.53	14.39	14.60
QCI		Cambodia	Age-standardized	Acute lymphoid leukemia	1990	9.39	9.26	9.45
QCI		Indonesia	Age-standardized	Acute lymphoid leukemia	1990	11.94	11.81	12.00
QCI		Lao People's Democratic Republic	Age-standardized	Acute lymphoid leukemia	1990	8.33	8.20	8.37
QCI		Malaysia	Age-standardized	Acute lymphoid leukemia	1990	17.73	17.61	17.81
QCI		Maldives	Age-standardized	Acute lymphoid leukemia	1990	13.03	12.90	13.10
QCI		Mauritius	Age-standardized	Acute lymphoid leukemia	1990	26.51	26.38	26.61
QCI		Myanmar	Age-standardized	Acute lymphoid leukemia	1990	7.86	7.73	7.90
QCI		Philippines	Age-standardized	Acute lymphoid leukemia	1990	14.87	14.74	14.93
QCI		Seychelles	Age-standardized	Acute lymphoid leukemia	1990	21.64	21.49	21.74
QCI		Sri Lanka	Age-standardized	Acute lymphoid leukemia	1990	19.31	19.16	19.39
QCI		Thailand	Age-standardized	Acute lymphoid leukemia	1990	23.46	23.33	23.56
QCI		Timor-Leste	Age-standardized	Acute lymphoid leukemia	1990	8.16	8.04	8.21
QCI		Viet nam	Age-standardized	Acute lymphoid leukemia	1990	20.72	20.58	20.82
QCI	region	**Oceania**	Age-standardized	Acute lymphoid leukemia	1990	9.02	8.88	9.07
QCI		American Samoa	Age-standardized	Acute lymphoid leukemia	1990	23.10	22.93	23.25
QCI		Cook Islands	Age-standardized	Acute lymphoid leukemia	1990	28.93	28.78	29.06
QCI		Fiji	Age-standardized	Acute lymphoid leukemia	1990	10.79	10.65	10.85
QCI		Guam	Age-standardized	Acute lymphoid leukemia	1990	37.12	36.99	37.24
QCI		Kiribati	Age-standardized	Acute lymphoid leukemia	1990	2.58	2.45	2.61
QCI		Marshall Islands	Age-standardized	Acute lymphoid leukemia	1990	9.58	9.44	9.66
QCI		Micronesia (Federated States of)	Age-standardized	Acute lymphoid leukemia	1990	8.53	8.38	8.60
QCI		Northern Mariana Islands	Age-standardized	Acute lymphoid leukemia	1990	27.33	27.17	27.46
QCI		Nauru	Age-standardized	Acute lymphoid leukemia	1990	10.46	10.34	10.53
QCI		Niue	Age-standardized	Acute lymphoid leukemia	1990	18.06	17.92	18.16
QCI		Palau	Age-standardized	Acute lymphoid leukemia	1990	21.36	21.21	21.48
QCI		Papua New Guinea	Age-standardized	Acute lymphoid leukemia	1990	6.53	6.41	6.57
QCI		Samoa	Age-standardized	Acute lymphoid leukemia	1990	16.69	16.55	16.79
QCI		Solomon Islands	Age-standardized	Acute lymphoid leukemia	1990	7.40	7.27	7.45
QCI		Tokelau	Age-standardized	Acute lymphoid leukemia	1990	12.39	12.25	12.47
QCI		Tonga	Age-standardized	Acute lymphoid leukemia	1990	19.51	19.36	19.61
QCI		Tuvalu	Age-standardized	Acute lymphoid leukemia	1990	8.59	8.46	8.65
QCI		Vanuatu	Age-standardized	Acute lymphoid leukemia	1990	9.53	9.39	9.60
QCI	region	**North Africa and Middle East**	Age-standardized	Acute lymphoid leukemia	1990	18.28	18.15	18.36
QCI		Afghanistan	Age-standardized	Acute lymphoid leukemia	1990	13.87	13.74	13.94
QCI		Algeria	Age-standardized	Acute lymphoid leukemia	1990	15.81	15.69	15.87
QCI		Bahrain	Age-standardized	Acute lymphoid leukemia	1990	24.36	24.21	24.47

measure	group	location	age	cause	year	val	lower	upper
QCI		Egypt	Age-standardized	Acute lymphoid leukemia	1990	13.20	13.08	13.26
QCI		Iran (Islamic Republic of)	Age-standardized	Acute lymphoid leukemia	1990	24.08	23.95	24.17
QCI		Iraq	Age-standardized	Acute lymphoid leukemia	1990	17.27	17.15	17.34
QCI		Jordan	Age-standardized	Acute lymphoid leukemia	1990	24.02	23.90	24.11
QCI		Kuwait	Age-standardized	Acute lymphoid leukemia	1990	55.83	55.72	55.95
QCI		Lebanon	Age-standardized	Acute lymphoid leukemia	1990	27.60	27.46	27.72
QCI		Libya	Age-standardized	Acute lymphoid leukemia	1990	26.79	26.65	26.89
QCI		Morocco	Age-standardized	Acute lymphoid leukemia	1990	12.90	12.75	12.95
QCI		Oman	Age-standardized	Acute lymphoid leukemia	1990	27.80	27.67	27.90
QCI		Palestine	Age-standardized	Acute lymphoid leukemia	1990	22.55	22.42	22.62
QCI		Qatar	Age-standardized	Acute lymphoid leukemia	1990	28.27	28.12	28.38
QCI		Saudi Arabia	Age-standardized	Acute lymphoid leukemia	1990	18.31	18.18	18.38
QCI		Sudan	Age-standardized	Acute lymphoid leukemia	1990	10.22	10.09	10.27
QCI		Syrian Arab Republic	Age-standardized	Acute lymphoid leukemia	1990	18.60	18.47	18.68
QCI		Tunisia	Age-standardized	Acute lymphoid leukemia	1990	31.35	31.21	31.45
QCI		Turkey	Age-standardized	Acute lymphoid leukemia	1990	17.92	17.80	18.00
QCI		United Arab Emirates	Age-standardized	Acute lymphoid leukemia	1990	20.07	19.93	20.15
QCI		Yemen	Age-standardized	Acute lymphoid leukemia	1990	13.09	12.96	13.15
QCI	**region**	**South Asia**	Age-standardized	Acute lymphoid leukemia	1990	9.33	9.20	9.38
QCI		Bangladesh	Age-standardized	Acute lymphoid leukemia	1990	7.07	6.95	7.11
QCI		Bhutan	Age-standardized	Acute lymphoid leukemia	1990	10.06	9.93	10.11
QCI		India	Age-standardized	Acute lymphoid leukemia	1990	9.93	9.80	9.98
QCI		Nepal	Age-standardized	Acute lymphoid leukemia	1990	7.65	7.52	7.69
QCI		Pakistan	Age-standardized	Acute lymphoid leukemia	1990	9.13	9.01	9.18
QCI	**region**	**Southern Sub-Saharan Africa**	Age-standardized	Acute lymphoid leukemia	1990	17.84	17.70	17.92
QCI		Botswana	Age-standardized	Acute lymphoid leukemia	1990	16.14	16.00	16.22
QCI		Eswatini	Age-standardized	Acute lymphoid leukemia	1990	15.82	15.68	15.91
QCI		Lesotho	Age-standardized	Acute lymphoid leukemia	1990	15.69	15.54	15.77
QCI		Namibia	Age-standardized	Acute lymphoid leukemia	1990	14.87	14.73	14.95
QCI		South Africa	Age-standardized	Acute lymphoid leukemia	1990	18.31	18.18	18.40
QCI		Zimbabwe	Age-standardized	Acute lymphoid leukemia	1990	17.56	17.42	17.65
QCI	**region**	**Western Sub-Saharan Africa**	Age-standardized	Acute lymphoid leukemia	1990	2.45	2.33	2.46
QCI		Benin	Age-standardized	Acute lymphoid leukemia	1990	2.75	2.64	2.76
QCI		Burkina Faso	Age-standardized	Acute lymphoid leukemia	1990	4.04	3.93	4.06
QCI		Cabo Verde	Age-standardized	Acute lymphoid leukemia	1990	14.72	14.60	14.77
QCI		Cameroon	Age-standardized	Acute lymphoid leukemia	1990	5.53	5.42	5.55
QCI		Chad	Age-standardized	Acute lymphoid leukemia	1990	2.27	2.16	2.29
QCI		Coted'Ivoire	Age-standardized	Acute lymphoid leukemia	1990	5.00	4.89	5.02
QCI		Gambia	Age-standardized	Acute lymphoid leukemia	1990	6.29	6.18	6.31
QCI		Ghana	Age-standardized	Acute lymphoid leukemia	1990	5.95	5.83	5.98
QCI		Guinea	Age-standardized	Acute lymphoid leukemia	1990	0.55	0.43	0.55
QCI		Guinea-Bissau	Age-standardized	Acute lymphoid leukemia	1990	2.76	2.64	2.77
QCI		Liberia	Age-standardized	Acute lymphoid leukemia	1990	1.26	1.15	1.27
QCI		Mali	Age-standardized	Acute lymphoid leukemia	1990	3.37	3.26	3.39
QCI		Mauritania	Age-standardized	Acute lymphoid leukemia	1990	5.22	5.10	5.24
QCI		Niger	Age-standardized	Acute lymphoid leukemia	1990	0.00	0.00	0.00
QCI		Nigeria	Age-standardized	Acute lymphoid leukemia	1990	0.26	0.14	0.26
QCI		Sao Tome and Principe	Age-standardized	Acute lymphoid leukemia	1990	6.29	6.18	6.31
QCI		Senegal	Age-standardized	Acute lymphoid leukemia	1990	4.15	4.03	4.16
QCI		Sierra Leone	Age-standardized	Acute lymphoid leukemia	1990	1.70	1.59	1.70
QCI		Togo	Age-standardized	Acute lymphoid leukemia	1990	5.55	5.43	5.57
QCI	**region**	**Eastern Sub-Saharan Africa**	Age-standardized	Acute lymphoid leukemia	1990	8.79	8.65	8.83
QCI		Burundi	Age-standardized	Acute lymphoid leukemia	1990	4.70	4.59	4.73
QCI		Comoros	Age-standardized	Acute lymphoid leukemia	1990	7.19	7.08	7.22
QCI		Djibouti	Age-standardized	Acute lymphoid leukemia	1990	7.73	7.61	7.76
QCI		Eritrea	Age-standardized	Acute lymphoid leukemia	1990	6.40	6.28	6.43
QCI		Ethiopia	Age-standardized	Acute lymphoid leukemia	1990	9.99	9.86	10.05
QCI		Kenya	Age-standardized	Acute lymphoid leukemia	1990	14.09	13.96	14.15
QCI		Madagascar	Age-standardized	Acute lymphoid leukemia	1990	6.21	6.10	6.24
QCI		Malawi	Age-standardized	Acute lymphoid leukemia	1990	2.52	2.40	2.52
QCI		Mozambique	Age-standardized	Acute lymphoid leukemia	1990	2.29	2.17	2.30
QCI		Rwanda	Age-standardized	Acute lymphoid leukemia	1990	4.85	4.73	4.87
QCI		Somalia	Age-standardized	Acute lymphoid leukemia	1990	7.72	7.59	7.76
QCI		South Sudan	Age-standardized	Acute lymphoid leukemia	1990	6.84	6.72	6.87
QCI		Uganda	Age-standardized	Acute lymphoid leukemia	1990	6.74	6.62	6.77
QCI		United Republic of Tanzania	Age-standardized	Acute lymphoid leukemia	1990	6.66	6.55	6.69
QCI		Zambia	Age-standardized	Acute lymphoid leukemia	1990	5.65	5.53	5.67
QCI	**region**	**Central Sub-Saharan Africa**	Age-standardized	Acute lymphoid leukemia	1990	9.07	8.94	9.12
QCI		Angola	Age-standardized	Acute lymphoid leukemia	1990	7.58	7.46	7.62
QCI		Central African Republic	Age-standardized	Acute lymphoid leukemia	1990	9.07	8.94	9.12
QCI		Congo	Age-standardized	Acute lymphoid leukemia	1990	11.69	11.56	11.75
QCI		Democratic Republic of the Congo	Age-standardized	Acute lymphoid leukemia	1990	9.26	9.14	9.31
QCI		Equatorial Guinea	Age-standardized	Acute lymphoid leukemia	1990	9.38	9.26	9.43
QCI		Gabon	Age-standardized	Acute lymphoid leukemia	1990	13.29	13.15	13.35
QCI	**Global**	**Global**	Age-standardized	Acute lymphoid leukemia	2021	58.46	58.36	58.58
QCI	**SDI**	**Low SDI**	Age-standardized	Acute lymphoid leukemia	2021	14.10	13.97	14.16
QCI	**SDI**	**Low-middle SDI**	Age-standardized	Acute lymphoid leukemia	2021	24.34	24.22	24.44
QCI	**SDI**	**Middle SDI**	Age-standardized	Acute lymphoid leukemia	2021	57.47	57.35	57.59
QCI	**SDI**	**High-middle SDI**	Age-standardized	Acute lymphoid leukemia	2021	82.68	82.62	82.77

measure	group	location	age	cause	year	val	lower	upper
QCI	**SDI**	**High SDI**	Age-standardized	Acute lymphoid leukemia	2021	90.75	90.72	90.85
QCI	**region**	**High-income North America**	Age-standardized	Acute lymphoid leukemia	2021	87.74	87.69	87.83
QCI		Canada	Age-standardized	Acute lymphoid leukemia	2021	94.64	94.62	94.74
QCI		Greenland	Age-standardized	Acute lymphoid leukemia	2021	51.89	51.75	52.02
QCI		United States of America	Age-standardized	Acute lymphoid leukemia	2021	86.82	86.77	86.91
QCI	**region**	**Australasia**	Age-standardized	Acute lymphoid leukemia	2021	92.01	91.98	92.10
QCI		Australia	Age-standardized	Acute lymphoid leukemia	2021	92.29	92.27	92.39
QCI		New Zealand	Age-standardized	Acute lymphoid leukemia	2021	90.59	90.56	90.69
QCI	**region**	**High-income Asia Pacific**	Age-standardized	Acute lymphoid leukemia	2021	93.82	93.80	93.92
QCI		Brunei Darussalam	Age-standardized	Acute lymphoid leukemia	2021	44.29	44.17	44.42
QCI		Japan	Age-standardized	Acute lymphoid leukemia	2021	94.14	94.12	94.24
QCI		Republic of Korea	Age-standardized	Acute lymphoid leukemia	2021	93.60	93.58	93.70
QCI		Singapore	Age-standardized	Acute lymphoid leukemia	2021	91.93	91.90	92.02
QCI	**region**	**Western Europe**	Age-standardized	Acute lymphoid leukemia	2021	96.17	96.15	96.27
QCI		Andorra	Age-standardized	Acute lymphoid leukemia	2021	97.07	97.06	97.17
QCI		Austria	Age-standardized	Acute lymphoid leukemia	2021	96.24	96.22	96.34
QCI		Belgium	Age-standardized	Acute lymphoid leukemia	2021	96.13	96.12	96.23
QCI		Cyprus	Age-standardized	Acute lymphoid leukemia	2021	91.76	91.73	91.86
QCI		Denmark	Age-standardized	Acute lymphoid leukemia	2021	96.28	96.26	96.38
QCI		Finland	Age-standardized	Acute lymphoid leukemia	2021	92.90	92.87	92.99
QCI		France	Age-standardized	Acute lymphoid leukemia	2021	96.09	96.08	96.19
QCI		Germany	Age-standardized	Acute lymphoid leukemia	2021	96.17	96.16	96.27
QCI		Greece	Age-standardized	Acute lymphoid leukemia	2021	93.97	93.95	94.07
QCI		Iceland	Age-standardized	Acute lymphoid leukemia	2021	96.05	96.04	96.15
QCI		Ireland	Age-standardized	Acute lymphoid leukemia	2021	95.82	95.80	95.92
QCI		Israel	Age-standardized	Acute lymphoid leukemia	2021	92.08	92.05	92.18
QCI		Italy	Age-standardized	Acute lymphoid leukemia	2021	97.23	97.22	97.32
QCI		Luxembourg	Age-standardized	Acute lymphoid leukemia	2021	96.78	96.77	96.88
QCI		Malta	Age-standardized	Acute lymphoid leukemia	2021	96.38	96.37	96.48
QCI		Monaco	Age-standardized	Acute lymphoid leukemia	2021	99.94	99.94	100.00
QCI		Netherlands	Age-standardized	Acute lymphoid leukemia	2021	95.39	95.37	95.49
QCI		Norway	Age-standardized	Acute lymphoid leukemia	2021	95.37	95.36	95.47
QCI		Portugal	Age-standardized	Acute lymphoid leukemia	2021	94.30	94.28	94.39
QCI		San Marino	Age-standardized	Acute lymphoid leukemia	2021	97.04	97.03	97.14
QCI		Spain	Age-standardized	Acute lymphoid leukemia	2021	97.98	97.98	98.08
QCI		Sweden	Age-standardized	Acute lymphoid leukemia	2021	97.75	97.74	97.85
QCI		Switzerland	Age-standardized	Acute lymphoid leukemia	2021	99.80	99.80	99.91
QCI		United Kingdom	Age-standardized	Acute lymphoid leukemia	2021	95.28	95.27	95.38
QCI	**region**	**Southern Latin America**	Age-standardized	Acute lymphoid leukemia	2021	55.07	54.95	55.20
QCI		Argentina	Age-standardized	Acute lymphoid leukemia	2021	49.13	49.00	49.25
QCI		Chile	Age-standardized	Acute lymphoid leukemia	2021	67.70	67.60	67.83
QCI		Uruguay	Age-standardized	Acute lymphoid leukemia	2021	54.54	54.43	54.66
QCI	**region**	**Eastern Europe**	Age-standardized	Acute lymphoid leukemia	2021	65.60	65.50	65.71
QCI		Belarus	Age-standardized	Acute lymphoid leukemia	2021	71.85	71.75	71.98
QCI		Estonia	Age-standardized	Acute lymphoid leukemia	2021	79.18	79.12	79.29
QCI		Latvia	Age-standardized	Acute lymphoid leukemia	2021	64.55	64.44	64.67
QCI		Lithuania	Age-standardized	Acute lymphoid leukemia	2021	64.01	63.90	64.14
QCI		Republic of Moldova	Age-standardized	Acute lymphoid leukemia	2021	58.23	58.10	58.35
QCI		Russian Federation	Age-standardized	Acute lymphoid leukemia	2021	67.33	67.22	67.45
QCI		Ukraine	Age-standardized	Acute lymphoid leukemia	2021	57.49	57.38	57.62
QCI	**region**	**Central Europe**	Age-standardized	Acute lymphoid leukemia	2021	67.82	67.71	67.94
QCI		Albania	Age-standardized	Acute lymphoid leukemia	2021	71.40	71.32	71.51
QCI		Bosnia and Herzegovina	Age-standardized	Acute lymphoid leukemia	2021	54.70	54.57	54.84
QCI		Bulgaria	Age-standardized	Acute lymphoid leukemia	2021	50.55	50.43	50.67
QCI		Croatia	Age-standardized	Acute lymphoid leukemia	2021	83.18	83.12	83.28
QCI		Czechia	Age-standardized	Acute lymphoid leukemia	2021	75.91	75.83	76.02
QCI		Hungary	Age-standardized	Acute lymphoid leukemia	2021	70.14	70.04	70.26
QCI		Montenegro	Age-standardized	Acute lymphoid leukemia	2021	67.09	66.99	67.21
QCI		North Macedonia	Age-standardized	Acute lymphoid leukemia	2021	52.47	52.33	52.61
QCI		Poland	Age-standardized	Acute lymphoid leukemia	2021	69.75	69.65	69.86
QCI		Romania	Age-standardized	Acute lymphoid leukemia	2021	58.87	58.75	58.99
QCI		Serbia	Age-standardized	Acute lymphoid leukemia	2021	61.93	61.81	62.06
QCI		Slovakia	Age-standardized	Acute lymphoid leukemia	2021	70.36	70.28	70.48
QCI		Slovenia	Age-standardized	Acute lymphoid leukemia	2021	83.77	83.71	83.88
QCI	**region**	**Central Asia**	Age-standardized	Acute lymphoid leukemia	2021	35.38	35.25	35.48
QCI		Armenia	Age-standardized	Acute lymphoid leukemia	2021	54.42	54.30	54.54
QCI		Azerbaijan	Age-standardized	Acute lymphoid leukemia	2021	40.96	40.84	41.06
QCI		Georgia	Age-standardized	Acute lymphoid leukemia	2021	38.44	38.30	38.57
QCI		Kazakhstan	Age-standardized	Acute lymphoid leukemia	2021	46.77	46.64	46.90
QCI		Kyrgyzstan	Age-standardized	Acute lymphoid leukemia	2021	38.55	38.43	38.67
QCI		Mongolia	Age-standardized	Acute lymphoid leukemia	2021	25.44	25.31	25.54
QCI		Tajikistan	Age-standardized	Acute lymphoid leukemia	2021	22.33	22.22	22.41
QCI		Turkmenistan	Age-standardized	Acute lymphoid leukemia	2021	27.22	27.09	27.31
QCI		Uzbekistan	Age-standardized	Acute lymphoid leukemia	2021	32.90	32.79	33.00
QCI	**region**	**Central Latin America**	Age-standardized	Acute lymphoid leukemia	2021	38.96	38.83	39.08
QCI		Colombia	Age-standardized	Acute lymphoid leukemia	2021	51.17	51.06	51.28
QCI		Costa Rica	Age-standardized	Acute lymphoid leukemia	2021	56.76	56.65	56.89
QCI		El Salvador	Age-standardized	Acute lymphoid leukemia	2021	37.99	37.86	38.10
QCI		Guatemala	Age-standardized	Acute lymphoid leukemia	2021	23.07	22.93	23.15

measure	group	location	age	cause	year	val	lower	upper
QCI		Honduras	Age-standardized	Acute lymphoid leukemia	2021	21.72	21.58	21.83
QCI		Mexico	Age-standardized	Acute lymphoid leukemia	2021	36.54	36.41	36.65
QCI		Nicaragua	Age-standardized	Acute lymphoid leukemia	2021	36.30	36.17	36.42
QCI		Panama	Age-standardized	Acute lymphoid leukemia	2021	53.44	53.33	53.56
QCI		Venezuela (Bolivarian Republic of)	Age-standardized	Acute lymphoid leukemia	2021	38.90	38.78	39.01
QCI	**region**	**Andean Latin America**	Age-standardized	Acute lymphoid leukemia	2021	41.97	41.84	42.08
QCI		Bolivia (Plurinational State of)	Age-standardized	Acute lymphoid leukemia	2021	21.86	21.73	21.95
QCI		Ecuador	Age-standardized	Acute lymphoid leukemia	2021	35.41	35.27	35.53
QCI		Peru	Age-standardized	Acute lymphoid leukemia	2021	51.32	51.20	51.44
QCI	**region**	**Caribbean**	Age-standardized	Acute lymphoid leukemia	2021	27.28	27.15	27.36
QCI		Antigua and Barbuda	Age-standardized	Acute lymphoid leukemia	2021	54.18	54.06	54.30
QCI		Bahamas	Age-standardized	Acute lymphoid leukemia	2021	32.86	32.73	32.96
QCI		Barbados	Age-standardized	Acute lymphoid leukemia	2021	50.47	50.35	50.58
QCI		Bermuda	Age-standardized	Acute lymphoid leukemia	2021	96.54	96.53	96.64
QCI		Belize	Age-standardized	Acute lymphoid leukemia	2021	32.03	31.90	32.14
QCI		Cuba	Age-standardized	Acute lymphoid leukemia	2021	63.04	62.94	63.16
QCI		Dominica	Age-standardized	Acute lymphoid leukemia	2021	28.83	28.70	28.94
QCI		Dominican Republic	Age-standardized	Acute lymphoid leukemia	2021	28.26	28.14	28.35
QCI		Grenada	Age-standardized	Acute lymphoid leukemia	2021	34.20	34.07	34.30
QCI		Guyana	Age-standardized	Acute lymphoid leukemia	2021	18.55	18.42	18.63
QCI		Haiti	Age-standardized	Acute lymphoid leukemia	2021	6.97	6.85	7.01
QCI		Jamaica	Age-standardized	Acute lymphoid leukemia	2021	43.90	43.77	44.01
QCI		Puerto Rico	Age-standardized	Acute lymphoid leukemia	2021	69.92	69.83	70.06
QCI		Saint Kitts and Nevis	Age-standardized	Acute lymphoid leukemia	2021	38.84	38.73	38.94
QCI		Saint Lucia	Age-standardized	Acute lymphoid leukemia	2021	35.63	35.50	35.73
QCI		Saint Vincent and the Grenadines	Age-standardized	Acute lymphoid leukemia	2021	26.73	26.60	26.81
QCI		Suriname	Age-standardized	Acute lymphoid leukemia	2021	22.51	22.38	22.59
QCI		Trinidad and Tobago	Age-standardized	Acute lymphoid leukemia	2021	36.26	36.13	36.38
QCI		United States Virgin Islands	Age-standardized	Acute lymphoid leukemia	2021	48.89	48.78	49.00
QCI	**region**	**Tropical Latin America**	Age-standardized	Acute lymphoid leukemia	2021	38.03	37.90	38.14
QCI		Brazil	Age-standardized	Acute lymphoid leukemia	2021	37.87	37.75	37.99
QCI		Paraguay	Age-standardized	Acute lymphoid leukemia	2021	39.99	39.86	40.10
QCI	**region**	**East Asia**	Age-standardized	Acute lymphoid leukemia	2021	81.64	81.58	81.73
QCI		China	Age-standardized	Acute lymphoid leukemia	2021	82.00	81.95	82.09
QCI		Democratic People's Republic of Korea	Age-standardized	Acute lymphoid leukemia	2021	38.91	38.77	39.03
QCI		Taiwan (Province of China)	Age-standardized	Acute lymphoid leukemia	2021	83.71	83.65	83.82
QCI	**region**	**Southeast Asia**	Age-standardized	Acute lymphoid leukemia	2021	31.12	30.98	31.23
QCI		Cambodia	Age-standardized	Acute lymphoid leukemia	2021	20.51	20.37	20.60
QCI		Indonesia	Age-standardized	Acute lymphoid leukemia	2021	23.81	23.67	23.90
QCI		Lao People's Democratic Republic	Age-standardized	Acute lymphoid leukemia	2021	15.75	15.62	15.82
QCI		Malaysia	Age-standardized	Acute lymphoid leukemia	2021	36.79	36.65	36.91
QCI		Maldives	Age-standardized	Acute lymphoid leukemia	2021	61.97	61.87	62.09
QCI		Mauritius	Age-standardized	Acute lymphoid leukemia	2021	48.09	47.97	48.21
QCI		Myanmar	Age-standardized	Acute lymphoid leukemia	2021	21.02	20.89	21.11
QCI		Philippines	Age-standardized	Acute lymphoid leukemia	2021	24.36	24.23	24.45
QCI		Seychelles	Age-standardized	Acute lymphoid leukemia	2021	37.66	37.51	37.80
QCI		Sri Lanka	Age-standardized	Acute lymphoid leukemia	2021	48.21	48.08	48.35
QCI		Thailand	Age-standardized	Acute lymphoid leukemia	2021	60.39	60.28	60.51
QCI		Timor-Leste	Age-standardized	Acute lymphoid leukemia	2021	17.49	17.35	17.57
QCI		Viet nam	Age-standardized	Acute lymphoid leukemia	2021	46.73	46.61	46.86
QCI	**region**	**Oceania**	Age-standardized	Acute lymphoid leukemia	2021	11.36	11.23	11.41
QCI		American Samoa	Age-standardized	Acute lymphoid leukemia	2021	30.49	30.32	30.64
QCI		Cook Islands	Age-standardized	Acute lymphoid leukemia	2021	68.06	67.95	68.19
QCI		Fiji	Age-standardized	Acute lymphoid leukemia	2021	19.26	19.12	19.36
QCI		Guam	Age-standardized	Acute lymphoid leukemia	2021	68.19	68.10	68.29
QCI		Kiribati	Age-standardized	Acute lymphoid leukemia	2021	6.68	6.55	6.74
QCI		Marshall Islands	Age-standardized	Acute lymphoid leukemia	2021	12.22	12.08	12.30
QCI		Micronesia (Federated States of)	Age-standardized	Acute lymphoid leukemia	2021	15.55	15.40	15.65
QCI		Northern Mariana Islands	Age-standardized	Acute lymphoid leukemia	2021	35.15	34.99	35.30
QCI		Nauru	Age-standardized	Acute lymphoid leukemia	2021	17.21	17.07	17.30
QCI		Niue	Age-standardized	Acute lymphoid leukemia	2021	41.85	41.74	41.95
QCI		Palau	Age-standardized	Acute lymphoid leukemia	2021	25.06	24.90	25.19
QCI		Papua New Guinea	Age-standardized	Acute lymphoid leukemia	2021	9.33	9.20	9.38
QCI		Samoa	Age-standardized	Acute lymphoid leukemia	2021	27.06	26.92	27.19
QCI		Solomon Islands	Age-standardized	Acute lymphoid leukemia	2021	11.77	11.63	11.85
QCI		Tokelau	Age-standardized	Acute lymphoid leukemia	2021	41.17	41.06	41.27
QCI		Tonga	Age-standardized	Acute lymphoid leukemia	2021	30.72	30.58	30.85
QCI		Tuvalu	Age-standardized	Acute lymphoid leukemia	2021	17.87	17.71	17.97
QCI		Vanuatu	Age-standardized	Acute lymphoid leukemia	2021	11.84	11.70	11.93
QCI	**region**	**North Africa and Middle East**	Age-standardized	Acute lymphoid leukemia	2021	43.77	43.64	43.89
QCI		Afghanistan	Age-standardized	Acute lymphoid leukemia	2021	16.88	16.74	16.97
QCI		Algeria	Age-standardized	Acute lymphoid leukemia	2021	53.47	53.36	53.59
QCI		Bahrain	Age-standardized	Acute lymphoid leukemia	2021	59.75	59.63	59.88
QCI		Egypt	Age-standardized	Acute lymphoid leukemia	2021	35.85	35.70	35.97
QCI		Iran (Islamic Republic of)	Age-standardized	Acute lymphoid leukemia	2021	57.04	56.93	57.17
QCI		Iraq	Age-standardized	Acute lymphoid leukemia	2021	55.23	55.12	55.35
QCI		Jordan	Age-standardized	Acute lymphoid leukemia	2021	68.15	68.06	68.26
QCI		Kuwait	Age-standardized	Acute lymphoid leukemia	2021	85.25	85.20	85.35
QCI		Lebanon	Age-standardized	Acute lymphoid leukemia	2021	66.90	66.80	67.03

measure	group	location	age	cause	year	val	lower	upper
QCI		Libya	Age-standardized	Acute lymphoid leukemia	2021	48.14	48.01	48.27
QCI		Morocco	Age-standardized	Acute lymphoid leukemia	2021	32.74	32.61	32.86
QCI		Oman	Age-standardized	Acute lymphoid leukemia	2021	69.89	69.81	70.01
QCI		Palestine	Age-standardized	Acute lymphoid leukemia	2021	53.34	53.22	53.46
QCI		Qatar	Age-standardized	Acute lymphoid leukemia	2021	75.15	75.08	75.27
QCI		Saudi Arabia	Age-standardized	Acute lymphoid leukemia	2021	51.00	50.87	51.13
QCI		Sudan	Age-standardized	Acute lymphoid leukemia	2021	27.06	26.92	27.16
QCI		Syrian Arab Republic	Age-standardized	Acute lymphoid leukemia	2021	50.42	50.29	50.54
QCI		Tunisia	Age-standardized	Acute lymphoid leukemia	2021	61.84	61.73	61.96
QCI		Turkey	Age-standardized	Acute lymphoid leukemia	2021	64.14	64.04	64.26
QCI		United Arab Emirates	Age-standardized	Acute lymphoid leukemia	2021	41.73	41.60	41.86
QCI		Yemen	Age-standardized	Acute lymphoid leukemia	2021	21.23	21.09	21.32
QCI	**region**	**South Asia**	Age-standardized	Acute lymphoid leukemia	2021	21.94	21.80	22.03
QCI		Bangladesh	Age-standardized	Acute lymphoid leukemia	2021	24.27	24.14	24.36
QCI		Bhutan	Age-standardized	Acute lymphoid leukemia	2021	25.14	25.00	25.24
QCI		India	Age-standardized	Acute lymphoid leukemia	2021	24.36	24.22	24.46
QCI		Nepal	Age-standardized	Acute lymphoid leukemia	2021	19.60	19.48	19.68
QCI		Pakistan	Age-standardized	Acute lymphoid leukemia	2021	13.91	13.77	13.97
QCI	**region**	**Southern Sub-Saharan Africa**	Age-standardized	Acute lymphoid leukemia	2021	21.08	20.95	21.18
QCI		Botswana	Age-standardized	Acute lymphoid leukemia	2021	19.91	19.77	20.00
QCI		Eswatini	Age-standardized	Acute lymphoid leukemia	2021	17.58	17.45	17.67
QCI		Lesotho	Age-standardized	Acute lymphoid leukemia	2021	15.96	15.82	16.04
QCI		Namibia	Age-standardized	Acute lymphoid leukemia	2021	21.98	21.84	22.07
QCI		South Africa	Age-standardized	Acute lymphoid leukemia	2021	24.85	24.72	24.95
QCI		Zimbabwe	Age-standardized	Acute lymphoid leukemia	2021	15.63	15.49	15.71
QCI	**region**	**Western Sub-Saharan Africa**	Age-standardized	Acute lymphoid leukemia	2021	10.59	10.48	10.63
QCI		Benin	Age-standardized	Acute lymphoid leukemia	2021	10.73	10.61	10.77
QCI		Burkina Faso	Age-standardized	Acute lymphoid leukemia	2021	8.38	8.26	8.41
QCI		Cabo Verde	Age-standardized	Acute lymphoid leukemia	2021	33.79	33.67	33.89
QCI		Cameroon	Age-standardized	Acute lymphoid leukemia	2021	12.61	12.48	12.65
QCI		Chad	Age-standardized	Acute lymphoid leukemia	2021	5.23	5.12	5.25
QCI		Coted'Ivoire	Age-standardized	Acute lymphoid leukemia	2021	13.18	13.05	13.22
QCI		Gambia	Age-standardized	Acute lymphoid leukemia	2021	14.64	14.52	14.70
QCI		Ghana	Age-standardized	Acute lymphoid leukemia	2021	15.52	15.40	15.57
QCI		Guinea	Age-standardized	Acute lymphoid leukemia	2021	7.22	7.10	7.24
QCI		Guinea-Bissau	Age-standardized	Acute lymphoid leukemia	2021	8.39	8.27	8.42
QCI		Liberia	Age-standardized	Acute lymphoid leukemia	2021	12.92	12.80	12.97
QCI		Mali	Age-standardized	Acute lymphoid leukemia	2021	10.54	10.41	10.58
QCI		Mauritania	Age-standardized	Acute lymphoid leukemia	2021	23.62	23.50	23.70
QCI		Niger	Age-standardized	Acute lymphoid leukemia	2021	6.96	6.84	6.99
QCI		Nigeria	Age-standardized	Acute lymphoid leukemia	2021	10.21	10.10	10.24
QCI		Sao Tome and Principe	Age-standardized	Acute lymphoid leukemia	2021	21.56	21.44	21.63
QCI		Senegal	Age-standardized	Acute lymphoid leukemia	2021	12.96	12.84	13.01
QCI		Sierra Leone	Age-standardized	Acute lymphoid leukemia	2021	9.92	9.80	9.96
QCI		Togo	Age-standardized	Acute lymphoid leukemia	2021	13.00	12.87	13.05
QCI	**region**	**Eastern Sub-Saharan Africa**	Age-standardized	Acute lymphoid leukemia	2021	14.38	14.25	14.44
QCI		Burundi	Age-standardized	Acute lymphoid leukemia	2021	10.31	10.18	10.36
QCI		Comoros	Age-standardized	Acute lymphoid leukemia	2021	13.39	13.27	13.45
QCI		Djibouti	Age-standardized	Acute lymphoid leukemia	2021	15.28	15.16	15.35
QCI		Eritrea	Age-standardized	Acute lymphoid leukemia	2021	10.79	10.65	10.84
QCI		Ethiopia	Age-standardized	Acute lymphoid leukemia	2021	15.58	15.45	15.65
QCI		Kenya	Age-standardized	Acute lymphoid leukemia	2021	18.22	18.08	18.31
QCI		Madagascar	Age-standardized	Acute lymphoid leukemia	2021	11.66	11.54	11.71
QCI		Malawi	Age-standardized	Acute lymphoid leukemia	2021	12.58	12.46	12.64
QCI		Mozambique	Age-standardized	Acute lymphoid leukemia	2021	10.43	10.31	10.48
QCI		Rwanda	Age-standardized	Acute lymphoid leukemia	2021	14.42	14.29	14.48
QCI		Somalia	Age-standardized	Acute lymphoid leukemia	2021	11.37	11.24	11.43
QCI		South Sudan	Age-standardized	Acute lymphoid leukemia	2021	10.84	10.72	10.88
QCI		Uganda	Age-standardized	Acute lymphoid leukemia	2021	13.80	13.68	13.85
QCI		United Republic of Tanzania	Age-standardized	Acute lymphoid leukemia	2021	14.59	14.47	14.64
QCI		Zambia	Age-standardized	Acute lymphoid leukemia	2021	12.87	12.75	12.92
QCI	**region**	**Central Sub-Saharan Africa**	Age-standardized	Acute lymphoid leukemia	2021	15.02	14.89	15.10
QCI		Angola	Age-standardized	Acute lymphoid leukemia	2021	14.83	14.70	14.90
QCI		Central African Republic	Age-standardized	Acute lymphoid leukemia	2021	11.04	10.90	11.09
QCI		Congo	Age-standardized	Acute lymphoid leukemia	2021	16.93	16.80	17.01
QCI		Democratic Republic of the Congo	Age-standardized	Acute lymphoid leukemia	2021	15.07	14.93	15.14
QCI		Equatorial Guinea	Age-standardized	Acute lymphoid leukemia	2021	22.92	22.79	23.01
QCI		Gabon	Age-standardized	Acute lymphoid leukemia	2021	21.04	20.90	21.12

Table S6. Gender disparity ratio (GDR) of Quality of Care Index (QCI) for acute lymphoblastic leukemia in 204 countries and territories in 1990 and 2021.

measure	group	location	age	cause	year	val
GDR	**Global**	**Global**	Age-standardized	Acute lymphoid leukemia	1990	0.96
GDR	**SDI**	**Low SDI**	Age-standardized	Acute lymphoid leukemia	1990	1.02
GDR	**SDI**	**Low-middle SDI**	Age-standardized	Acute lymphoid leukemia	1990	0.76
GDR	**SDI**	**Middle SDI**	Age-standardized	Acute lymphoid leukemia	1990	0.99
GDR	**SDI**	**High-middle SDI**	Age-standardized	Acute lymphoid leukemia	1990	0.99
GDR	**SDI**	**High SDI**	Age-standardized	Acute lymphoid leukemia	1990	1.02
GDR	**region**	**High-income North America**	Age-standardized	Acute lymphoid leukemia	1990	1.02
GDR		Canada	Age-standardized	Acute lymphoid leukemia	1990	1.04
GDR		Greenland	Age-standardized	Acute lymphoid leukemia	1990	0.92
GDR		United States of America	Age-standardized	Acute lymphoid leukemia	1990	1.02
GDR	**region**	**Australasia**	Age-standardized	Acute lymphoid leukemia	1990	1.04
GDR		Australia	Age-standardized	Acute lymphoid leukemia	1990	1.04
GDR		New Zealand	Age-standardized	Acute lymphoid leukemia	1990	0.99
GDR	**region**	**High-income Asia Pacific**	Age-standardized	Acute lymphoid leukemia	1990	1.04
GDR		Brunei Darussalam	Age-standardized	Acute lymphoid leukemia	1990	1.16
GDR		Japan	Age-standardized	Acute lymphoid leukemia	1990	1.03
GDR		Republic of Korea	Age-standardized	Acute lymphoid leukemia	1990	1.04
GDR		Singapore	Age-standardized	Acute lymphoid leukemia	1990	1.00
GDR	**region**	**Western Europe**	Age-standardized	Acute lymphoid leukemia	1990	1.03
GDR		Andorra	Age-standardized	Acute lymphoid leukemia	1990	1.07
GDR		Austria	Age-standardized	Acute lymphoid leukemia	1990	1.06
GDR		Belgium	Age-standardized	Acute lymphoid leukemia	1990	1.05
GDR		Cyprus	Age-standardized	Acute lymphoid leukemia	1990	1.08
GDR		Denmark	Age-standardized	Acute lymphoid leukemia	1990	0.99
GDR		Finland	Age-standardized	Acute lymphoid leukemia	1990	1.16
GDR		France	Age-standardized	Acute lymphoid leukemia	1990	1.07
GDR		Germany	Age-standardized	Acute lymphoid leukemia	1990	1.08
GDR		Greece	Age-standardized	Acute lymphoid leukemia	1990	1.05
GDR		Iceland	Age-standardized	Acute lymphoid leukemia	1990	1.01
GDR		Ireland	Age-standardized	Acute lymphoid leukemia	1990	1.03
GDR		Israel	Age-standardized	Acute lymphoid leukemia	1990	1.10
GDR		Italy	Age-standardized	Acute lymphoid leukemia	1990	1.03
GDR		Luxembourg	Age-standardized	Acute lymphoid leukemia	1990	1.08
GDR		Malta	Age-standardized	Acute lymphoid leukemia	1990	1.03
GDR		Monaco	Age-standardized	Acute lymphoid leukemia	1990	1.09
GDR		Netherlands	Age-standardized	Acute lymphoid leukemia	1990	1.03
GDR		Norway	Age-standardized	Acute lymphoid leukemia	1990	1.04
GDR		Portugal	Age-standardized	Acute lymphoid leukemia	1990	1.27
GDR		San Marino	Age-standardized	Acute lymphoid leukemia	1990	1.01
GDR		Spain	Age-standardized	Acute lymphoid leukemia	1990	1.06
GDR		Sweden	Age-standardized	Acute lymphoid leukemia	1990	1.04
GDR		Switzerland	Age-standardized	Acute lymphoid leukemia	1990	0.99
GDR		United Kingdom	Age-standardized	Acute lymphoid leukemia	1990	0.96
GDR	**region**	**Southern Latin America**	Age-standardized	Acute lymphoid leukemia	1990	1.11
GDR		Argentina	Age-standardized	Acute lymphoid leukemia	1990	1.12
GDR		Chile	Age-standardized	Acute lymphoid leukemia	1990	1.08
GDR		Uruguay	Age-standardized	Acute lymphoid leukemia	1990	1.09
GDR	**region**	**Eastern Europe**	Age-standardized	Acute lymphoid leukemia	1990	0.72
GDR		Belarus	Age-standardized	Acute lymphoid leukemia	1990	0.69
GDR		Estonia	Age-standardized	Acute lymphoid leukemia	1990	0.70
GDR		Latvia	Age-standardized	Acute lymphoid leukemia	1990	0.74
GDR		Lithuania	Age-standardized	Acute lymphoid leukemia	1990	0.73
GDR		Republic of Moldova	Age-standardized	Acute lymphoid leukemia	1990	0.65
GDR		Russian Federation	Age-standardized	Acute lymphoid leukemia	1990	0.71
GDR		Ukraine	Age-standardized	Acute lymphoid leukemia	1990	0.74
GDR	**region**	**Central Europe**	Age-standardized	Acute lymphoid leukemia	1990	0.59
GDR		Albania	Age-standardized	Acute lymphoid leukemia	1990	0.48
GDR		Bosnia and Herzegovina	Age-standardized	Acute lymphoid leukemia	1990	0.60
GDR		Bulgaria	Age-standardized	Acute lymphoid leukemia	1990	0.57
GDR		Croatia	Age-standardized	Acute lymphoid leukemia	1990	0.67
GDR		Czechia	Age-standardized	Acute lymphoid leukemia	1990	0.65
GDR		Hungary	Age-standardized	Acute lymphoid leukemia	1990	0.59
GDR		Montenegro	Age-standardized	Acute lymphoid leukemia	1990	0.72
GDR		North Macedonia	Age-standardized	Acute lymphoid leukemia	1990	0.57
GDR		Poland	Age-standardized	Acute lymphoid leukemia	1990	0.62
GDR		Romania	Age-standardized	Acute lymphoid leukemia	1990	0.50
GDR		Serbia	Age-standardized	Acute lymphoid leukemia	1990	0.54
GDR		Slovakia	Age-standardized	Acute lymphoid leukemia	1990	0.63
GDR		Slovenia	Age-standardized	Acute lymphoid leukemia	1990	0.67
GDR	**region**	**Central Asia**	Age-standardized	Acute lymphoid leukemia	1990	0.60
GDR		Armenia	Age-standardized	Acute lymphoid leukemia	1990	0.67
GDR		Azerbaijan	Age-standardized	Acute lymphoid leukemia	1990	0.47
GDR		Georgia	Age-standardized	Acute lymphoid leukemia	1990	0.65
GDR		Kazakhstan	Age-standardized	Acute lymphoid leukemia	1990	0.61
GDR		Kyrgyzstan	Age-standardized	Acute lymphoid leukemia	1990	0.55
GDR		Mongolia	Age-standardized	Acute lymphoid leukemia	1990	0.57
GDR		Tajikistan	Age-standardized	Acute lymphoid leukemia	1990	0.50
GDR		Turkmenistan	Age-standardized	Acute lymphoid leukemia	1990	0.54
GDR		Uzbekistan	Age-standardized	Acute lymphoid leukemia	1990	0.60
GDR	**region**	**Central Latin America**	Age-standardized	Acute lymphoid leukemia	1990	0.96
GDR		Colombia	Age-standardized	Acute lymphoid leukemia	1990	0.99
GDR		Costa Rica	Age-standardized	Acute lymphoid leukemia	1990	1.00
GDR		El Salvador	Age-standardized	Acute lymphoid leukemia	1990	0.89
GDR		Guatemala	Age-standardized	Acute lymphoid leukemia	1990	0.67
GDR		Honduras	Age-standardized	Acute lymphoid leukemia	1990	0.79
GDR		Mexico	Age-standardized	Acute lymphoid leukemia	1990	0.95
GDR		Nicaragua	Age-standardized	Acute lymphoid leukemia	1990	0.97

measure	group	location	age	cause	year	val
GDR		Panama	Age-standardized	Acute lymphoid leukemia	1990	1.06
GDR		Venezuela (Bolivarian Republic of)	Age-standardized	Acute lymphoid leukemia	1990	1.08
GDR	**region**	**Andean Latin America**	Age-standardized	Acute lymphoid leukemia	1990	0.89
GDR		Bolivia (Plurinational State of)	Age-standardized	Acute lymphoid leukemia	1990	0.86
GDR		Ecuador	Age-standardized	Acute lymphoid leukemia	1990	0.95
GDR		Peru	Age-standardized	Acute lymphoid leukemia	1990	0.92
GDR	**region**	**Caribbean**	Age-standardized	Acute lymphoid leukemia	1990	0.82
GDR		Antigua and Barbuda	Age-standardized	Acute lymphoid leukemia	1990	1.15
GDR		Bahamas	Age-standardized	Acute lymphoid leukemia	1990	1.18
GDR		Barbados	Age-standardized	Acute lymphoid leukemia	1990	1.20
GDR		Bermuda	Age-standardized	Acute lymphoid leukemia	1990	1.74
GDR		Belize	Age-standardized	Acute lymphoid leukemia	1990	1.21
GDR		Cuba	Age-standardized	Acute lymphoid leukemia	1990	1.10
GDR		Dominica	Age-standardized	Acute lymphoid leukemia	1990	0.95
GDR		Dominican Republic	Age-standardized	Acute lymphoid leukemia	1990	0.91
GDR		Grenada	Age-standardized	Acute lymphoid leukemia	1990	0.95
GDR		Guyana	Age-standardized	Acute lymphoid leukemia	1990	1.25
GDR		Haiti	Age-standardized	Acute lymphoid leukemia	1990	0.43
GDR		Jamaica	Age-standardized	Acute lymphoid leukemia	1990	1.13
GDR		Puerto Rico	Age-standardized	Acute lymphoid leukemia	1990	1.29
GDR		Saint Kitts and Nevis	Age-standardized	Acute lymphoid leukemia	1990	1.02
GDR		Saint Lucia	Age-standardized	Acute lymphoid leukemia	1990	1.14
GDR		Saint Vincent and the Grenadines	Age-standardized	Acute lymphoid leukemia	1990	1.04
GDR		Suriname	Age-standardized	Acute lymphoid leukemia	1990	0.74
GDR		Trinidad and Tobago	Age-standardized	Acute lymphoid leukemia	1990	0.95
GDR		United States Virgin Islands	Age-standardized	Acute lymphoid leukemia	1990	1.22
GDR	**region**	**Tropical Latin America**	Age-standardized	Acute lymphoid leukemia	1990	0.98
GDR		Brazil	Age-standardized	Acute lymphoid leukemia	1990	0.97
GDR		Paraguay	Age-standardized	Acute lymphoid leukemia	1990	1.06
GDR	**region**	**East Asia**	Age-standardized	Acute lymphoid leukemia	1990	1.05
GDR		China	Age-standardized	Acute lymphoid leukemia	1990	1.05
GDR		Democratic People's Republic of Korea	Age-standardized	Acute lymphoid leukemia	1990	1.00
GDR		Taiwan (Province of China)	Age-standardized	Acute lymphoid leukemia	1990	1.09
GDR	**region**	**Southeast Asia**	Age-standardized	Acute lymphoid leukemia	1990	0.86
GDR		Cambodia	Age-standardized	Acute lymphoid leukemia	1990	0.58
GDR		Indonesia	Age-standardized	Acute lymphoid leukemia	1990	0.73
GDR		Lao People's Democratic Republic	Age-standardized	Acute lymphoid leukemia	1990	0.57
GDR		Malaysia	Age-standardized	Acute lymphoid leukemia	1990	0.93
GDR		Maldives	Age-standardized	Acute lymphoid leukemia	1990	0.72
GDR		Mauritius	Age-standardized	Acute lymphoid leukemia	1990	1.07
GDR		Myanmar	Age-standardized	Acute lymphoid leukemia	1990	0.58
GDR		Philippines	Age-standardized	Acute lymphoid leukemia	1990	0.91
GDR		Seychelles	Age-standardized	Acute lymphoid leukemia	1990	1.16
GDR		Sri Lanka	Age-standardized	Acute lymphoid leukemia	1990	1.09
GDR		Thailand	Age-standardized	Acute lymphoid leukemia	1990	1.05
GDR		Timor-Leste	Age-standardized	Acute lymphoid leukemia	1990	0.51
GDR		Viet Nam	Age-standardized	Acute lymphoid leukemia	1990	0.98
GDR	**region**	**Oceania**	Age-standardized	Acute lymphoid leukemia	1990	0.46
GDR		American Samoa	Age-standardized	Acute lymphoid leukemia	1990	0.95
GDR		Cook Islands	Age-standardized	Acute lymphoid leukemia	1990	0.85
GDR		Fiji	Age-standardized	Acute lymphoid leukemia	1990	0.61
GDR		Guam	Age-standardized	Acute lymphoid leukemia	1990	1.24
GDR		Kiribati	Age-standardized	Acute lymphoid leukemia	1990	0.00
GDR		Marshall Islands	Age-standardized	Acute lymphoid leukemia	1990	0.70
GDR		Micronesia (Federated States of)	Age-standardized	Acute lymphoid leukemia	1990	0.76
GDR		Nauru	Age-standardized	Acute lymphoid leukemia	1990	0.86
GDR		Northern Mariana Islands	Age-standardized	Acute lymphoid leukemia	1990	0.54
GDR		Niue	Age-standardized	Acute lymphoid leukemia	1990	0.78
GDR		Palau	Age-standardized	Acute lymphoid leukemia	1990	0.89
GDR		Papua New Guinea	Age-standardized	Acute lymphoid leukemia	1990	0.54
GDR		Samoa	Age-standardized	Acute lymphoid leukemia	1990	0.84
GDR		Solomon Islands	Age-standardized	Acute lymphoid leukemia	1990	0.77
GDR		Tokelau	Age-standardized	Acute lymphoid leukemia	1990	0.73
GDR		Tonga	Age-standardized	Acute lymphoid leukemia	1990	0.75
GDR		Tuvalu	Age-standardized	Acute lymphoid leukemia	1990	0.93
GDR		Vanuatu	Age-standardized	Acute lymphoid leukemia	1990	0.77
GDR	**region**	**North Africa and Middle East**	Age-standardized	Acute lymphoid leukemia	1990	1.02
GDR		Afghanistan	Age-standardized	Acute lymphoid leukemia	1990	1.29
GDR		Algeria	Age-standardized	Acute lymphoid leukemia	1990	0.93
GDR		Bahrain	Age-standardized	Acute lymphoid leukemia	1990	1.02
GDR		Egypt	Age-standardized	Acute lymphoid leukemia	1990	1.02
GDR		Iran (Islamic Republic of)	Age-standardized	Acute lymphoid leukemia	1990	1.02
GDR		Iraq	Age-standardized	Acute lymphoid leukemia	1990	1.06
GDR		Jordan	Age-standardized	Acute lymphoid leukemia	1990	1.05
GDR		Kuwait	Age-standardized	Acute lymphoid leukemia	1990	1.07
GDR		Lebanon	Age-standardized	Acute lymphoid leukemia	1990	1.01
GDR		Libya	Age-standardized	Acute lymphoid leukemia	1990	1.04
GDR		Morocco	Age-standardized	Acute lymphoid leukemia	1990	1.01
GDR		Oman	Age-standardized	Acute lymphoid leukemia	1990	0.96
GDR		Palestine	Age-standardized	Acute lymphoid leukemia	1990	1.12
GDR		Qatar	Age-standardized	Acute lymphoid leukemia	1990	1.02
GDR		Saudi Arabia	Age-standardized	Acute lymphoid leukemia	1990	0.94
GDR		Sudan	Age-standardized	Acute lymphoid leukemia	1990	1.03
GDR		Syrian Arab Republic	Age-standardized	Acute lymphoid leukemia	1990	1.04
GDR		Tunisia	Age-standardized	Acute lymphoid leukemia	1990	1.07
GDR		Turkey	Age-standardized	Acute lymphoid leukemia	1990	1.11
GDR		United Arab Emirates	Age-standardized	Acute lymphoid leukemia	1990	1.07
GDR		Yemen	Age-standardized	Acute lymphoid leukemia	1990	1.03

measure	group	location	age	cause	year	val
GDR	**region**	**South Asia**	Age-standardized	Acute lymphoid leukemia	1990	0.59
GDR		Bangladesh	Age-standardized	Acute lymphoid leukemia	1990	0.45
GDR		Bhutan	Age-standardized	Acute lymphoid leukemia	1990	0.70
GDR		India	Age-standardized	Acute lymphoid leukemia	1990	0.60
GDR		Nepal	Age-standardized	Acute lymphoid leukemia	1990	0.63
GDR		Pakistan	Age-standardized	Acute lymphoid leukemia	1990	0.64
GDR	**region**	**Southern Sub-Saharan Africa**	Age-standardized	Acute lymphoid leukemia	1990	0.79
GDR		Botswana	Age-standardized	Acute lymphoid leukemia	1990	0.67
GDR		Eswatini	Age-standardized	Acute lymphoid leukemia	1990	0.65
GDR		Lesotho	Age-standardized	Acute lymphoid leukemia	1990	0.58
GDR		Namibia	Age-standardized	Acute lymphoid leukemia	1990	0.62
GDR		South Africa	Age-standardized	Acute lymphoid leukemia	1990	0.82
GDR		Zimbabwe	Age-standardized	Acute lymphoid leukemia	1990	0.72
GDR	**region**	**Western Sub-Saharan Africa**	Age-standardized	Acute lymphoid leukemia	1990	1.19
GDR		Benin	Age-standardized	Acute lymphoid leukemia	1990	1.01
GDR		Burkina Faso	Age-standardized	Acute lymphoid leukemia	1990	1.29
GDR		Cabo Verde	Age-standardized	Acute lymphoid leukemia	1990	0.78
GDR		Cameroon	Age-standardized	Acute lymphoid leukemia	1990	1.34
GDR		Chad	Age-standardized	Acute lymphoid leukemia	1990	1.84
GDR		Coted'Ivoire	Age-standardized	Acute lymphoid leukemia	1990	0.98
GDR		Gambia	Age-standardized	Acute lymphoid leukemia	1990	0.95
GDR		Ghana	Age-standardized	Acute lymphoid leukemia	1990	1.33
GDR		Guinea	Age-standardized	Acute lymphoid leukemia	1990	177.52
GDR		Guinea-Bissau	Age-standardized	Acute lymphoid leukemia	1990	6.52
GDR		Liberia	Age-standardized	Acute lymphoid leukemia	1990	1.36
GDR		Mali	Age-standardized	Acute lymphoid leukemia	1990	2.83
GDR		Mauritania	Age-standardized	Acute lymphoid leukemia	1990	1.30
GDR		Niger	Age-standardized	Acute lymphoid leukemia	1990	1.39
GDR		Nigeria	Age-standardized	Acute lymphoid leukemia	1990	0.20
GDR		Sao Tome and Principe	Age-standardized	Acute lymphoid leukemia	1990	1.19
GDR		Senegal	Age-standardized	Acute lymphoid leukemia	1990	1.09
GDR		Sierra Leone	Age-standardized	Acute lymphoid leukemia	1990	0.87
GDR		Togo	Age-standardized	Acute lymphoid leukemia	1990	1.20
GDR	**region**	**Eastern Sub-Saharan Africa**	Age-standardized	Acute lymphoid leukemia	1990	1.25
GDR		Burundi	Age-standardized	Acute lymphoid leukemia	1990	0.46
GDR		Comoros	Age-standardized	Acute lymphoid leukemia	1990	0.76
GDR		Djibouti	Age-standardized	Acute lymphoid leukemia	1990	0.70
GDR		Eritrea	Age-standardized	Acute lymphoid leukemia	1990	0.70
GDR		Ethiopia	Age-standardized	Acute lymphoid leukemia	1990	1.65
GDR		Kenya	Age-standardized	Acute lymphoid leukemia	1990	0.68
GDR		Madagascar	Age-standardized	Acute lymphoid leukemia	1990	0.67
GDR		Malawi	Age-standardized	Acute lymphoid leukemia	1990	0.28
GDR		Mozambique	Age-standardized	Acute lymphoid leukemia	1990	0.12
GDR		Rwanda	Age-standardized	Acute lymphoid leukemia	1990	0.67
GDR		Somalia	Age-standardized	Acute lymphoid leukemia	1990	0.63
GDR		South Sudan	Age-standardized	Acute lymphoid leukemia	1990	0.49
GDR		Uganda	Age-standardized	Acute lymphoid leukemia	1990	0.32
GDR		United Republic of Tanzania	Age-standardized	Acute lymphoid leukemia	1990	0.63
GDR		Zambia	Age-standardized	Acute lymphoid leukemia	1990	0.58
GDR	**region**	**Central Sub-Saharan Africa**	Age-standardized	Acute lymphoid leukemia	1990	0.35
GDR		Angola	Age-standardized	Acute lymphoid leukemia	1990	0.27
GDR		Central African Republic	Age-standardized	Acute lymphoid leukemia	1990	0.34
GDR		Congo	Age-standardized	Acute lymphoid leukemia	1990	0.63
GDR		Democratic Republic of the Congo	Age-standardized	Acute lymphoid leukemia	1990	0.34
GDR		Equatorial Guinea	Age-standardized	Acute lymphoid leukemia	1990	0.37
GDR		Gabon	Age-standardized	Acute lymphoid leukemia	1990	0.61
GDR	**Global**	**Global**	Age-standardized	Acute lymphoid leukemia	2021	1.00
GDR	**SDI**	**Low SDI**	Age-standardized	Acute lymphoid leukemia	2021	0.93
GDR	**SDI**	**Low-middle SDI**	Age-standardized	Acute lymphoid leukemia	2021	0.97
GDR	**SDI**	**Middle SDI**	Age-standardized	Acute lymphoid leukemia	2021	1.01
GDR	**SDI**	**High-middle SDI**	Age-standardized	Acute lymphoid leukemia	2021	1.01
GDR	**SDI**	**High SDI**	Age-standardized	Acute lymphoid leukemia	2021	1.00
GDR	**region**	**High-income North America**	Age-standardized	Acute lymphoid leukemia	2021	1.02
GDR		Canada	Age-standardized	Acute lymphoid leukemia	2021	1.01
GDR		Greenland	Age-standardized	Acute lymphoid leukemia	2021	0.99
GDR		United States of America	Age-standardized	Acute lymphoid leukemia	2021	1.02
GDR	**region**	**Australasia**	Age-standardized	Acute lymphoid leukemia	2021	1.02
GDR		Australia	Age-standardized	Acute lymphoid leukemia	2021	1.02
GDR		New Zealand	Age-standardized	Acute lymphoid leukemia	2021	1.02
GDR	**region**	**High-income Asia Pacific**	Age-standardized	Acute lymphoid leukemia	2021	1.00
GDR		Brunei Darussalam	Age-standardized	Acute lymphoid leukemia	2021	0.87
GDR		Japan	Age-standardized	Acute lymphoid leukemia	2021	1.00
GDR		Republic of Korea	Age-standardized	Acute lymphoid leukemia	2021	1.00
GDR		Singapore	Age-standardized	Acute lymphoid leukemia	2021	1.02
GDR	**region**	**Western Europe**	Age-standardized	Acute lymphoid leukemia	2021	1.00
GDR		Andorra	Age-standardized	Acute lymphoid leukemia	2021	1.02
GDR		Austria	Age-standardized	Acute lymphoid leukemia	2021	1.00
GDR		Belgium	Age-standardized	Acute lymphoid leukemia	2021	0.99
GDR		Cyprus	Age-standardized	Acute lymphoid leukemia	2021	1.01
GDR		Denmark	Age-standardized	Acute lymphoid leukemia	2021	0.98
GDR		Finland	Age-standardized	Acute lymphoid leukemia	2021	1.02
GDR		France	Age-standardized	Acute lymphoid leukemia	2021	1.02
GDR		Germany	Age-standardized	Acute lymphoid leukemia	2021	1.01
GDR		Greece	Age-standardized	Acute lymphoid leukemia	2021	1.02
GDR		Iceland	Age-standardized	Acute lymphoid leukemia	2021	1.03
GDR		Ireland	Age-standardized	Acute lymphoid leukemia	2021	0.97
GDR		Israel	Age-standardized	Acute lymphoid leukemia	2021	1.01
GDR		Italy	Age-standardized	Acute lymphoid leukemia	2021	1.00

measure	group	location	age	cause	year	val
GDR		Luxembourg	Age-standardized	Acute lymphoid leukemia	2021	0.97
GDR		Malta	Age-standardized	Acute lymphoid leukemia	2021	0.99
GDR		Monaco	Age-standardized	Acute lymphoid leukemia	2021	0.98
GDR		Netherlands	Age-standardized	Acute lymphoid leukemia	2021	1.00
GDR		Norway	Age-standardized	Acute lymphoid leukemia	2021	0.99
GDR		Portugal	Age-standardized	Acute lymphoid leukemia	2021	1.03
GDR		San Marino	Age-standardized	Acute lymphoid leukemia	2021	1.01
GDR		Spain	Age-standardized	Acute lymphoid leukemia	2021	1.02
GDR		Sweden	Age-standardized	Acute lymphoid leukemia	2021	1.01
GDR		Switzerland	Age-standardized	Acute lymphoid leukemia	2021	0.99
GDR		United Kingdom	Age-standardized	Acute lymphoid leukemia	2021	0.96
GDR	**region**	**Southern Latin America**	Age-standardized	Acute lymphoid leukemia	2021	1.06
GDR		Argentina	Age-standardized	Acute lymphoid leukemia	2021	1.06
GDR		Chile	Age-standardized	Acute lymphoid leukemia	2021	1.04
GDR		Uruguay	Age-standardized	Acute lymphoid leukemia	2021	1.06
GDR	**region**	**Eastern Europe**	Age-standardized	Acute lymphoid leukemia	2021	0.89
GDR		Belarus	Age-standardized	Acute lymphoid leukemia	2021	0.84
GDR		Estonia	Age-standardized	Acute lymphoid leukemia	2021	0.92
GDR		Latvia	Age-standardized	Acute lymphoid leukemia	2021	0.88
GDR		Lithuania	Age-standardized	Acute lymphoid leukemia	2021	0.87
GDR		Republic of Moldova	Age-standardized	Acute lymphoid leukemia	2021	0.85
GDR		Russian Federation	Age-standardized	Acute lymphoid leukemia	2021	0.90
GDR		Ukraine	Age-standardized	Acute lymphoid leukemia	2021	0.90
GDR	**region**	**Central Europe**	Age-standardized	Acute lymphoid leukemia	2021	0.77
GDR		Albania	Age-standardized	Acute lymphoid leukemia	2021	0.93
GDR		Bosnia and Herzegovina	Age-standardized	Acute lymphoid leukemia	2021	0.65
GDR		Bulgaria	Age-standardized	Acute lymphoid leukemia	2021	0.71
GDR		Croatia	Age-standardized	Acute lymphoid leukemia	2021	0.82
GDR		Czechia	Age-standardized	Acute lymphoid leukemia	2021	0.77
GDR		Hungary	Age-standardized	Acute lymphoid leukemia	2021	0.75
GDR		Montenegro	Age-standardized	Acute lymphoid leukemia	2021	0.74
GDR		North Macedonia	Age-standardized	Acute lymphoid leukemia	2021	0.57
GDR		Poland	Age-standardized	Acute lymphoid leukemia	2021	0.77
GDR		Romania	Age-standardized	Acute lymphoid leukemia	2021	0.74
GDR		Serbia	Age-standardized	Acute lymphoid leukemia	2021	0.71
GDR		Slovakia	Age-standardized	Acute lymphoid leukemia	2021	0.72
GDR		Slovenia	Age-standardized	Acute lymphoid leukemia	2021	0.91
GDR	**region**	**Central Asia**	Age-standardized	Acute lymphoid leukemia	2021	0.75
GDR		Armenia	Age-standardized	Acute lymphoid leukemia	2021	0.87
GDR		Azerbaijan	Age-standardized	Acute lymphoid leukemia	2021	0.66
GDR		Georgia	Age-standardized	Acute lymphoid leukemia	2021	0.89
GDR		Kazakhstan	Age-standardized	Acute lymphoid leukemia	2021	0.80
GDR		Kyrgyzstan	Age-standardized	Acute lymphoid leukemia	2021	0.81
GDR		Mongolia	Age-standardized	Acute lymphoid leukemia	2021	0.80
GDR		Tajikistan	Age-standardized	Acute lymphoid leukemia	2021	0.64
GDR		Turkmenistan	Age-standardized	Acute lymphoid leukemia	2021	0.78
GDR		Uzbekistan	Age-standardized	Acute lymphoid leukemia	2021	0.73
GDR	**region**	**Central Latin America**	Age-standardized	Acute lymphoid leukemia	2021	1.03
GDR		Colombia	Age-standardized	Acute lymphoid leukemia	2021	0.96
GDR		Costa Rica	Age-standardized	Acute lymphoid leukemia	2021	0.98
GDR		El Salvador	Age-standardized	Acute lymphoid leukemia	2021	0.98
GDR		Guatemala	Age-standardized	Acute lymphoid leukemia	2021	0.97
GDR		Honduras	Age-standardized	Acute lymphoid leukemia	2021	1.02
GDR		Mexico	Age-standardized	Acute lymphoid leukemia	2021	1.06
GDR		Nicaragua	Age-standardized	Acute lymphoid leukemia	2021	0.97
GDR		Panama	Age-standardized	Acute lymphoid leukemia	2021	1.02
GDR		Venezuela (Bolivarian Republic of)	Age-standardized	Acute lymphoid leukemia	2021	1.13
GDR	**region**	**Andean Latin America**	Age-standardized	Acute lymphoid leukemia	2021	0.97
GDR		Bolivia (Plurinational State of)	Age-standardized	Acute lymphoid leukemia	2021	0.98
GDR		Ecuador	Age-standardized	Acute lymphoid leukemia	2021	0.99
GDR		Peru	Age-standardized	Acute lymphoid leukemia	2021	0.99
GDR	**region**	**Caribbean**	Age-standardized	Acute lymphoid leukemia	2021	0.76
GDR		Antigua and Barbuda	Age-standardized	Acute lymphoid leukemia	2021	1.15
GDR		Bahamas	Age-standardized	Acute lymphoid leukemia	2021	1.09
GDR		Barbados	Age-standardized	Acute lymphoid leukemia	2021	1.14
GDR		Bermuda	Age-standardized	Acute lymphoid leukemia	2021	1.49
GDR		Belize	Age-standardized	Acute lymphoid leukemia	2021	1.07
GDR		Cuba	Age-standardized	Acute lymphoid leukemia	2021	1.02
GDR		Dominica	Age-standardized	Acute lymphoid leukemia	2021	1.10
GDR		Dominican Republic	Age-standardized	Acute lymphoid leukemia	2021	1.08
GDR		Grenada	Age-standardized	Acute lymphoid leukemia	2021	1.05
GDR		Guyana	Age-standardized	Acute lymphoid leukemia	2021	0.90
GDR		Haiti	Age-standardized	Acute lymphoid leukemia	2021	0.84
GDR		Jamaica	Age-standardized	Acute lymphoid leukemia	2021	1.06
GDR		Puerto Rico	Age-standardized	Acute lymphoid leukemia	2021	1.10
GDR		Saint Kitts and Nevis	Age-standardized	Acute lymphoid leukemia	2021	1.31
GDR		Saint Lucia	Age-standardized	Acute lymphoid leukemia	2021	1.21
GDR		Saint Vincent and the Grenadines	Age-standardized	Acute lymphoid leukemia	2021	0.96
GDR		Suriname	Age-standardized	Acute lymphoid leukemia	2021	0.87
GDR		Trinidad and Tobago	Age-standardized	Acute lymphoid leukemia	2021	1.10
GDR		United States Virgin Islands	Age-standardized	Acute lymphoid leukemia	2021	1.60
GDR	**region**	**Tropical Latin America**	Age-standardized	Acute lymphoid leukemia	2021	1.07
GDR		Brazil	Age-standardized	Acute lymphoid leukemia	2021	1.07
GDR		Paraguay	Age-standardized	Acute lymphoid leukemia	2021	1.06
GDR	**region**	**East Asia**	Age-standardized	Acute lymphoid leukemia	2021	1.01
GDR		China	Age-standardized	Acute lymphoid leukemia	2021	1.02
GDR		Democratic People's Republic of Korea	Age-standardized	Acute lymphoid leukemia	2021	0.96
GDR		Taiwan (Province of China)	Age-standardized	Acute lymphoid leukemia	2021	1.03

measure	group	location	age	cause	year	val
GDR	**region**	**Southeast Asia**	Age-standardized	Acute lymphoid leukemia	2021	1.04
GDR		Cambodia	Age-standardized	Acute lymphoid leukemia	2021	1.00
GDR		Indonesia	Age-standardized	Acute lymphoid leukemia	2021	1.02
GDR		Lao People's Democratic Republic	Age-standardized	Acute lymphoid leukemia	2021	0.88
GDR		Malaysia	Age-standardized	Acute lymphoid leukemia	2021	1.03
GDR		Maldives	Age-standardized	Acute lymphoid leukemia	2021	1.08
GDR		Mauritius	Age-standardized	Acute lymphoid leukemia	2021	1.07
GDR		Myanmar	Age-standardized	Acute lymphoid leukemia	2021	1.00
GDR		Philippines	Age-standardized	Acute lymphoid leukemia	2021	1.02
GDR		Seychelles	Age-standardized	Acute lymphoid leukemia	2021	0.78
GDR		Sri Lanka	Age-standardized	Acute lymphoid leukemia	2021	1.13
GDR		Thailand	Age-standardized	Acute lymphoid leukemia	2021	1.07
GDR		Timor-Leste	Age-standardized	Acute lymphoid leukemia	2021	0.94
GDR		Viet Nam	Age-standardized	Acute lymphoid leukemia	2021	1.06
GDR	**region**	**Oceania**	Age-standardized	Acute lymphoid leukemia	2021	0.51
GDR		American Samoa	Age-standardized	Acute lymphoid leukemia	2021	1.01
GDR		Cook Islands	Age-standardized	Acute lymphoid leukemia	2021	0.82
GDR		Fiji	Age-standardized	Acute lymphoid leukemia	2021	0.92
GDR		Guam	Age-standardized	Acute lymphoid leukemia	2021	1.04
GDR		Kiribati	Age-standardized	Acute lymphoid leukemia	2021	0.78
GDR		Marshall Islands	Age-standardized	Acute lymphoid leukemia	2021	0.72
GDR		Micronesia (Federated States of)	Age-standardized	Acute lymphoid leukemia	2021	0.74
GDR		Nauru	Age-standardized	Acute lymphoid leukemia	2021	0.78
GDR		Niue	Age-standardized	Acute lymphoid leukemia	2021	0.94
GDR		Northern Mariana Islands	Age-standardized	Acute lymphoid leukemia	2021	0.68
GDR		Palau	Age-standardized	Acute lymphoid leukemia	2021	0.76
GDR		Papua New Guinea	Age-standardized	Acute lymphoid leukemia	2021	0.62
GDR		Samoa	Age-standardized	Acute lymphoid leukemia	2021	0.70
GDR		Solomon Islands	Age-standardized	Acute lymphoid leukemia	2021	0.75
GDR		Tokelau	Age-standardized	Acute lymphoid leukemia	2021	0.85
GDR		Tonga	Age-standardized	Acute lymphoid leukemia	2021	0.70
GDR		Tuvalu	Age-standardized	Acute lymphoid leukemia	2021	0.73
GDR		Vanuatu	Age-standardized	Acute lymphoid leukemia	2021	0.72
GDR	**region**	**North Africa and Middle East**	Age-standardized	Acute lymphoid leukemia	2021	0.90
GDR		Afghanistan	Age-standardized	Acute lymphoid leukemia	2021	1.16
GDR		Algeria	Age-standardized	Acute lymphoid leukemia	2021	0.99
GDR		Bahrain	Age-standardized	Acute lymphoid leukemia	2021	1.02
GDR		Egypt	Age-standardized	Acute lymphoid leukemia	2021	0.89
GDR		Iran (Islamic Republic of)	Age-standardized	Acute lymphoid leukemia	2021	1.01
GDR		Iraq	Age-standardized	Acute lymphoid leukemia	2021	0.95
GDR		Jordan	Age-standardized	Acute lymphoid leukemia	2021	1.06
GDR		Kuwait	Age-standardized	Acute lymphoid leukemia	2021	1.06
GDR		Lebanon	Age-standardized	Acute lymphoid leukemia	2021	0.99
GDR		Libya	Age-standardized	Acute lymphoid leukemia	2021	0.98
GDR		Morocco	Age-standardized	Acute lymphoid leukemia	2021	0.94
GDR		Oman	Age-standardized	Acute lymphoid leukemia	2021	1.09
GDR		Palestine	Age-standardized	Acute lymphoid leukemia	2021	0.97
GDR		Qatar	Age-standardized	Acute lymphoid leukemia	2021	0.95
GDR		Saudi Arabia	Age-standardized	Acute lymphoid leukemia	2021	0.88
GDR		Sudan	Age-standardized	Acute lymphoid leukemia	2021	0.92
GDR		Syrian Arab Republic	Age-standardized	Acute lymphoid leukemia	2021	0.92
GDR		Tunisia	Age-standardized	Acute lymphoid leukemia	2021	0.88
GDR		Turkey	Age-standardized	Acute lymphoid leukemia	2021	1.08
GDR		United Arab Emirates	Age-standardized	Acute lymphoid leukemia	2021	0.92
GDR		Yemen	Age-standardized	Acute lymphoid leukemia	2021	1.03
GDR	**region**	**South Asia**	Age-standardized	Acute lymphoid leukemia	2021	0.83
GDR		Bangladesh	Age-standardized	Acute lymphoid leukemia	2021	0.95
GDR		Bhutan	Age-standardized	Acute lymphoid leukemia	2021	0.99
GDR		India	Age-standardized	Acute lymphoid leukemia	2021	0.86
GDR		Nepal	Age-standardized	Acute lymphoid leukemia	2021	0.95
GDR		Pakistan	Age-standardized	Acute lymphoid leukemia	2021	0.76
GDR	**region**	**Southern Sub-Saharan Africa**	Age-standardized	Acute lymphoid leukemia	2021	0.92
GDR		Botswana	Age-standardized	Acute lymphoid leukemia	2021	0.91
GDR		Eswatini	Age-standardized	Acute lymphoid leukemia	2021	0.84
GDR		Lesotho	Age-standardized	Acute lymphoid leukemia	2021	0.80
GDR		Namibia	Age-standardized	Acute lymphoid leukemia	2021	0.97
GDR		South Africa	Age-standardized	Acute lymphoid leukemia	2021	1.03
GDR		Zimbabwe	Age-standardized	Acute lymphoid leukemia	2021	0.73
GDR	**region**	**Western Sub-Saharan Africa**	Age-standardized	Acute lymphoid leukemia	2021	0.80
GDR		Benin	Age-standardized	Acute lymphoid leukemia	2021	0.78
GDR		Burkina Faso	Age-standardized	Acute lymphoid leukemia	2021	0.84
GDR		Cabo Verde	Age-standardized	Acute lymphoid leukemia	2021	0.81
GDR		Cameroon	Age-standardized	Acute lymphoid leukemia	2021	0.82
GDR		Chad	Age-standardized	Acute lymphoid leukemia	2021	1.06
GDR		Coted'Ivoire	Age-standardized	Acute lymphoid leukemia	2021	0.82
GDR		Gambia	Age-standardized	Acute lymphoid leukemia	2021	0.92
GDR		Ghana	Age-standardized	Acute lymphoid leukemia	2021	0.77
GDR		Guinea	Age-standardized	Acute lymphoid leukemia	2021	1.06
GDR		Guinea-Bissau	Age-standardized	Acute lymphoid leukemia	2021	1.56
GDR		Liberia	Age-standardized	Acute lymphoid leukemia	2021	0.79
GDR		Mali	Age-standardized	Acute lymphoid leukemia	2021	0.92
GDR		Mauritania	Age-standardized	Acute lymphoid leukemia	2021	0.73
GDR		Niger	Age-standardized	Acute lymphoid leukemia	2021	0.93
GDR		Nigeria	Age-standardized	Acute lymphoid leukemia	2021	0.77
GDR		Sao Tome and Principe	Age-standardized	Acute lymphoid leukemia	2021	0.75
GDR		Senegal	Age-standardized	Acute lymphoid leukemia	2021	0.88
GDR		Sierra Leone	Age-standardized	Acute lymphoid leukemia	2021	0.85
GDR		Togo	Age-standardized	Acute lymphoid leukemia	2021	0.88

measure	group	location	age	cause	year	val
GDR	**region**	**Eastern Sub-Saharan Africa**	Age-standardized	Acute lymphoid leukemia	2021	0.96
GDR		Burundi	Age-standardized	Acute lymphoid leukemia	2021	0.75
GDR		Comoros	Age-standardized	Acute lymphoid leukemia	2021	0.82
GDR		Djibouti	Age-standardized	Acute lymphoid leukemia	2021	0.89
GDR		Eritrea	Age-standardized	Acute lymphoid leukemia	2021	0.92
GDR		Ethiopia	Age-standardized	Acute lymphoid leukemia	2021	1.05
GDR		Kenya	Age-standardized	Acute lymphoid leukemia	2021	0.83
GDR		Madagascar	Age-standardized	Acute lymphoid leukemia	2021	0.86
GDR		Malawi	Age-standardized	Acute lymphoid leukemia	2021	0.82
GDR		Mozambique	Age-standardized	Acute lymphoid leukemia	2021	0.91
GDR		Rwanda	Age-standardized	Acute lymphoid leukemia	2021	0.83
GDR		Somalia	Age-standardized	Acute lymphoid leukemia	2021	0.92
GDR		South Sudan	Age-standardized	Acute lymphoid leukemia	2021	0.78
GDR		Uganda	Age-standardized	Acute lymphoid leukemia	2021	0.69
GDR		United Republic of Tanzania	Age-standardized	Acute lymphoid leukemia	2021	0.84
GDR		Zambia	Age-standardized	Acute lymphoid leukemia	2021	0.93
GDR	**region**	**Central Sub-Saharan Africa**	Age-standardized	Acute lymphoid leukemia	2021	0.73
GDR		Angola	Age-standardized	Acute lymphoid leukemia	2021	0.74
GDR		Central African Republic	Age-standardized	Acute lymphoid leukemia	2021	0.61
GDR		Congo	Age-standardized	Acute lymphoid leukemia	2021	0.87
GDR		Democratic Republic of the Congo	Age-standardized	Acute lymphoid leukemia	2021	0.71
GDR		Equatorial Guinea	Age-standardized	Acute lymphoid leukemia	2021	0.90
GDR		Gabon	Age-standardized	Acute lymphoid leukemia	2021	0.90

Table S7. The Quality of Care Index (QCI) of top and bottom three countries to gender disparity ratio (GDR) in 1990 and 2021.

Measure	Gender	GDR top three countries			GDR bottom three countries		
QCI in 1990	Male	Guinea (0.01)	Guinea-Bissau (0.76)	Mali (1.43)	Nigeria (1.48)	Mozambique (4.20)	Kiribati (4.06)
	Female	Guinea (1.78)	Guinea-Bissau (4.98)	Mali (4.03)	Nigeria (0.29)	Mozambique (0.50)	Kiribati (0)
QCI in 2021	Male	United States Virgin Islands (39.89)	Guinea-Bissau (6.62)	Bermuda (63.99)	Papua New Guinea (12.13)	Central African Republic (13.34)	North Macedonia (59.01)
	Female	United States Virgin Islands (63.90)	Guinea-Bissau (10.35)	Bermuda (95.50)	Papua New Guinea (7.47)	Central African Republic (8.20)	North Macedonia (33.50)

Table S8. Quality of Care Index (QCI) for acute lymphoblastic leukemia across different age groups globally in 1990 and 2021.

measure	location	age	cause	year	val	lower	upper
QCI	Global	<5 years	Acute lymphoid leukemia	1990	50.70	49.52	50.76
QCI	Global	5-9 years	Acute lymphoid leukemia	1990	28.35	26.32	28.41
QCI	Global	10-14 years	Acute lymphoid leukemia	1990	26.11	23.98	26.17
QCI	Global	15-19 years	Acute lymphoid leukemia	1990	24.62	20.81	24.68
QCI	Global	20-24 years	Acute lymphoid leukemia	1990	24.85	19.23	24.90
QCI	Global	25-29 years	Acute lymphoid leukemia	1990	24.31	15.89	24.36
QCI	Global	30-34 years	Acute lymphoid leukemia	1990	19.33	12.20	19.37
QCI	Global	35-39 years	Acute lymphoid leukemia	1990	16.61	9.14	16.67
QCI	Global	40-44 years	Acute lymphoid leukemia	1990	15.10	9.05	15.16
QCI	Global	45-49 years	Acute lymphoid leukemia	1990	13.63	9.38	13.70
QCI	Global	50-54 years	Acute lymphoid leukemia	1990	15.61	9.44	15.67
QCI	Global	55-59 years	Acute lymphoid leukemia	1990	18.01	8.05	18.09
QCI	Global	60-64 years	Acute lymphoid leukemia	1990	19.13	8.70	19.28
QCI	Global	65-69 years	Acute lymphoid leukemia	1990	22.20	12.16	22.27
QCI	Global	70-74 years	Acute lymphoid leukemia	1990	36.95	22.16	37.13
QCI	Global	75-79 years	Acute lymphoid leukemia	1990	50.99	40.61	51.60
QCI	Global	80-84 years	Acute lymphoid leukemia	1990	46.57	46.27	46.75
QCI	Global	85-89 years	Acute lymphoid leukemia	1990	49.09	46.14	49.95
QCI	Global	90-94 years	Acute lymphoid leukemia	1990	55.18	54.47	55.41
QCI	Global	95+ years	Acute lymphoid leukemia	1990	19.73	17.76	23.69
QCI	Global	<5 years	Acute lymphoid leukemia	2021	84.47	84.09	84.50
QCI	Global	5-9 years	Acute lymphoid leukemia	2021	61.64	60.54	61.71
QCI	Global	10-14 years	Acute lymphoid leukemia	2021	55.15	53.83	55.22
QCI	Global	15-19 years	Acute lymphoid leukemia	2021	50.79	48.28	50.86
QCI	Global	20-24 years	Acute lymphoid leukemia	2021	47.24	43.27	47.30
QCI	Global	25-29 years	Acute lymphoid leukemia	2021	44.45	38.26	44.50
QCI	Global	30-34 years	Acute lymphoid leukemia	2021	41.38	36.21	41.44
QCI	Global	35-39 years	Acute lymphoid leukemia	2021	37.21	31.59	37.31
QCI	Global	40-44 years	Acute lymphoid leukemia	2021	33.52	28.77	33.64
QCI	Global	45-49 years	Acute lymphoid leukemia	2021	30.87	27.44	31.02
QCI	Global	50-54 years	Acute lymphoid leukemia	2021	31.25	26.21	31.34
QCI	Global	55-59 years	Acute lymphoid leukemia	2021	31.36	23.02	31.48
QCI	Global	60-64 years	Acute lymphoid leukemia	2021	30.05	21.02	30.13
QCI	Global	65-69 years	Acute lymphoid leukemia	2021	30.73	21.78	30.81
QCI	Global	70-74 years	Acute lymphoid leukemia	2021	49.20	37.28	49.44
QCI	Global	75-79 years	Acute lymphoid leukemia	2021	60.48	52.11	61.19
QCI	Global	80-84 years	Acute lymphoid leukemia	2021	50.36	50.07	50.56
QCI	Global	85-89 years	Acute lymphoid leukemia	2021	52.54	49.85	53.45
QCI	Global	90-94 years	Acute lymphoid leukemia	2021	58.77	58.12	59.03
QCI	Global	95+ years	Acute lymphoid leukemia	2021	24.20	22.20	29.21

Table S9. Gender disparity ratio (GDR) of acute lymphoblastic leukemia across different age groups globally in 1990 and 2021.

measure	location	age	cause	year	val
GDR	Global	<5	Acute lymphoid leukemia	1990	0.97
GDR	Global	5 to 9	Acute lymphoid leukemia	1990	1.05
GDR	Global	10 to 14	Acute lymphoid leukemia	1990	1.01
GDR	Global	15 to 19	Acute lymphoid leukemia	1990	0.86
GDR	Global	20 to 24	Acute lymphoid leukemia	1990	0.71
GDR	Global	25 to 29	Acute lymphoid leukemia	1990	0.56
GDR	Global	30 to 34	Acute lymphoid leukemia	1990	0.56
GDR	Global	35 to 39	Acute lymphoid leukemia	1990	0.56
GDR	Global	40 to 44	Acute lymphoid leukemia	1990	0.62
GDR	Global	45 to 49	Acute lymphoid leukemia	1990	0.70
GDR	Global	50 to 54	Acute lymphoid leukemia	1990	0.91
GDR	Global	55 to 59	Acute lymphoid leukemia	1990	0.99
GDR	Global	60 to 64	Acute lymphoid leukemia	1990	0.95
GDR	Global	65 to 69	Acute lymphoid leukemia	1990	0.96
GDR	Global	70 to 74	Acute lymphoid leukemia	1990	1.06
GDR	Global	75 to 79	Acute lymphoid leukemia	1990	1.05
GDR	Global	80 to 84	Acute lymphoid leukemia	1990	1.13
GDR	Global	85 to 89	Acute lymphoid leukemia	1990	1.44
GDR	Global	90 to 94	Acute lymphoid leukemia	1990	2.19
GDR	Global	95 plus	Acute lymphoid leukemia	1990	0.95
GDR	Global	<5	Acute lymphoid leukemia	2021	1.02
GDR	Global	5 to 9	Acute lymphoid leukemia	2021	1.03
GDR	Global	10 to 14	Acute lymphoid leukemia	2021	1.03
GDR	Global	15 to 19	Acute lymphoid leukemia	2021	0.93
GDR	Global	20 to 24	Acute lymphoid leukemia	2021	0.85
GDR	Global	25 to 29	Acute lymphoid leukemia	2021	0.78
GDR	Global	30 to 34	Acute lymphoid leukemia	2021	0.85
GDR	Global	35 to 39	Acute lymphoid leukemia	2021	0.87
GDR	Global	40 to 44	Acute lymphoid leukemia	2021	0.93
GDR	Global	45 to 49	Acute lymphoid leukemia	2021	1.06
GDR	Global	50 to 54	Acute lymphoid leukemia	2021	1.26
GDR	Global	55 to 59	Acute lymphoid leukemia	2021	1.28
GDR	Global	60 to 64	Acute lymphoid leukemia	2021	1.24
GDR	Global	65 to 69	Acute lymphoid leukemia	2021	1.17
GDR	Global	70 to 74	Acute lymphoid leukemia	2021	1.14
GDR	Global	75 to 79	Acute lymphoid leukemia	2021	1.06
GDR	Global	80 to 84	Acute lymphoid leukemia	2021	1.08
GDR	Global	85 to 89	Acute lymphoid leukemia	2021	1.40
GDR	Global	90 to 94	Acute lymphoid leukemia	2021	2.20
GDR	Global	95 plus	Acute lymphoid leukemia	2021	0.99

Table S10. Sensitivity analysis of the Quality of Care Index (QCI) using equal-weight calculation, sequential exclusion of indicators, and Min-Max normalization as alternatives to principal component analysis.

	Equal weights	Exclude MIR	Exclude YYR	Exclude DPR	Exclude PIR	Min-max normalization
Top 5 countries	Switzerland	Switzerland	Monaco	Monaco	Monaco	Switzerland
	Monaco	Monaco	Switzerland	Switzerland	Switzerland	Monaco
	Spain	Spain	Spain	Spain	Spain	Spain
	Italy	Sweden	Sweden	Sweden	Sweden	Italy
	Bermuda	Italy	Italy	Italy	Italy	Bermuda
Bottom 5 countries	Burkina Faso	Burkina Faso	Niger	Guinea	Kiribati	Burkina Faso
	Haiti	Haiti	Vanuatu	Haiti	Haiti	Haiti
	Niger	Niger	Haiti	Niger	Guinea	Niger
	Guinea	Guinea	Chad	Kiribati	Niger	Guinea
	Chad	Chad	Kiribati	Chad	Chad	Chad
